# Suppression of Alternative Lipooligosaccharide Glycosyltransferase Activity by UDP-Galactose Epimerase Enhances Murine Lung Infection and Evasion of Serum IgM

**DOI:** 10.3389/fcimb.2019.00160

**Published:** 2019-05-15

**Authors:** Sandy M. Wong, Mary Darby Jackson, Brian J. Akerley

**Affiliations:** Department of Microbiology and Immunology, University of Mississippi Medical Center, Jackson, MS, United States

**Keywords:** lipooligosaccharide (LOS), lung infection, *Haemophilus influenzae*, NTHi, Tn-seq, HITS, immune evasion

## Abstract

In pathogens that produce lipooligosaccharide (LOS), sugar residues within the surface-exposed LOS outer core mediate interactions with components of the host immune system, promoting bacterial infection. Many LOS structures are controlled by phase variation mediated by random slipped-strand base mispairing, which can reversibly switch gene expression on or off. Phase variation diversifies the LOS, however its adaptive role is not well-understood. Nontypeable *Haemophilus influenzae* (NTHi) is an important pathogen that causes a range of illnesses in the upper and lower respiratory tract. In NTHi a phase variable galactosyltransferase encoded by *lic2A* initiates galactose chain extension of the LOS outer core. The donor substrate for Lic2A, UDP-galactose, is generated from UDP-glucose by UDP-galactose epimerase encoded by *galE*. Our previous fitness profiling of *H. influenzae* mutants in a murine lung model showed that the *galE* mutant had a severe survival defect, while the *lic2A* mutant's defect was modest, leading us to postulate that unidentified factors act as suppressors of potential defects in a *lic2A* mutant. Herein we conducted a genome-wide genetic interaction screen to identify genes epistatic on *lic2A* for survival in the murine lung. An unexpected finding was that *galE* mutants exhibited restored virulence properties in a *lic2A* mutant background. We identified an alternative antibody epitope generated by Lic2A in the *galE* mutant that increased sensitivity to classical complement mediated killing in human serum. Deletion of *lic2A* or restoration of UDP-galactose synthesis alleviated the *galE* mutant's virulence defects. These studies indicate that when deprived of its galactosyl substrate, Lic2A acquires an alternative activity leading to increased recognition of NTHi by IgM and decreased survival in the lung model. Biofilm formation was increased by deletion of *galE* and by increased availability of UDP-GlcNAc precursors that can compete with UDP-galactose production. NTHi's ability to reversibly inactivate *lic2A* by phase-variation may influence survival in niches of infection in which UDP-Galactose levels are limiting.

## Introduction

Lipooligosaccharide (LOS), a short chain form of lipopolysaccharide (LPS) that lacks the long repetitive polysaccharide O-antigen of LPS, is the principal component of the outer-membrane of bacteria that produce it, mediating interactions with host immune defenses that determine the outcome of infection. *Haemophilus influenzae*, a Gram-negative bacterium that colonizes the human respiratory tract, causes a range of illnesses including otitis media, sinusitis, and exacerbation of chronic obstructive pulmonary disease (Klein, 1997; Murphy et al., 2004; Wiertsema et al., 2011; Ahearn et al., 2017). In *H. influenzae*, LOS structure varies between strains, yet several features are conserved such as lipid A, an inner core usually composed of a single 3-deoxy-D-*manno*-octulosonic acid linked to three heptose residues, and an outer core containing a short heteropolymer of glucose and galactose residues in different configurations extending from the heptosyl residues (Moxon and Murphy, 2000). Glucose and galactose extensions of the outer core often terminate in host-like structures including sialic acid, phosphorylcholine (PC), and *N*-acetylgalactosamine (Hood et al., 1999; Risberg et al., 1999) mediating numerous aspects of immune evasion. Sialylated LOS is critical for colonization of the middle ear and nasopharynx in a chinchilla model of otitis media (Bouchet et al., 2003) as well as in a gerbil middle ear infection model and a rat pulmonary model (Swords et al., 2004). PC modification of LOS promotes persistence in the respiratory tract in an infant rat model of infection (Weiser et al., 1998), and mutants deficient in the *N*-acetylgalactosamine structure exhibit reduced lethality in a mouse model of bacteremia (Hood et al., 1996).

**Figure 1:**
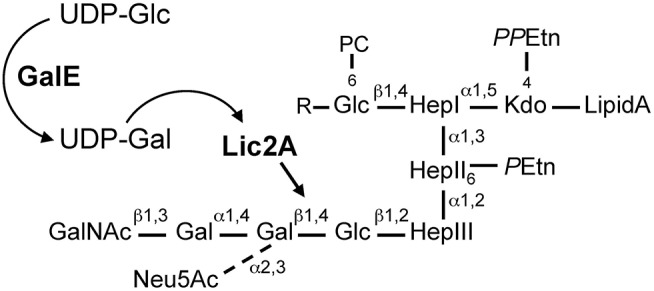
*H. influenzae* Rd LOS. GalE generates UDP-Gal for galactose extension by Lic2A. Diagram is based on structural information as described (Risberg et al., 1999; Hood et al., 2001, 2004b). R, tetrasaccharide extensions, Neu5Ac-α2,3-Gal-β1,4-GlcNAc-β1,3-Gal or *P*Etn-6-GalNAc-α1,6-Gal-β1,4-GlcNAc-β1,3-Gal. Glc, glucose; Gal, galactose; UDP-Glc, UDP-glucose; UDP-Gal, UDP-galactose; UDP, uridine diphosphate; Neu5Ac*, N*-acetylneuraminic acid; GalNAc, *N*-acetylgalactosamine; PC, phosphorylcholine; Hep, heptose; Kdo, 2-keto-3-deoxyoctulosonic acid; *PP*Etn, pyrophosphoethanolamine; *P*Etn, phosphoethanolamine.

In the *H. influenzae* Rd strain and NTHi strain 375, the *lic2A* encoded galactosyltransferase initiates galactose chain extension by addition of a β-galactose in a 1,4-linkage to a glucose appending from heptose III of the inner core (Hood et al., 1999, 2001). UDP-glucose 4-epimerase encoded by *galE* interconverts endogenous UDP-glucose (UDP-Glc) to UDP-galactose (UDP-Gal) (Frey, 1996), which then serves as donor of galactosyl units for biosynthesis of LOS outer core structures of *H. influenzae*, including the *lic2A* dependent extension (Maskell et al., 1992). From the glucose on Hep I, extensions containing several alternative “cryptic” LOS structures have also been identified that vary between strains and environmental conditions (Hood et al., 2004b; Figure 1). In our previous studies we utilized genome-wide mutant fitness analyses of *H. influenzae* Rd in the murine lung, a model of bacterial clearance that detects genes required for immune evasion and other determinants of host-pathogen interactions. Our approach, termed HITS (High-throughput Insertion Tracking by deep-Sequencing), revealed that mutations in *galE* conferred a severe survival defect relative to other potential virulence determinants. Surprisingly, mutations in *lic2A* conferred modest or insignificant defects (Gawronski et al., 2009; Wong et al., 2013). Because structural analysis of the major LOS species in Rd indicates that *lic2A* is required for addition of the initial galactose unit to a glucose extension in the outer core (Risberg et al., 1999; Hood et al., 2001), a similar LOS structure might be predicted for both the *galE* and *lic2A* mutants, in contrast to their very different *in vivo* survival levels identified by HITS in the murine model. It is possible that other LOS genes are functionally redundant with the *lic2A* gene during infection. For example, some cryptic LOS structures contain galactose, and therefore may contribute to the *galE* phenotype. As the cryptic structures vary under different environmental conditions and possess several alternative forms (Hood et al., 2004b), it is unclear which, if any, of them relate to these observations. We postulated that unidentified factors such as other complementary LOS genes or other genetic interactions act as suppressors of potential virulence defects in the *lic2A* mutant.

In this report, we undertook a functional genomics approach using a genetic interaction screen to identify genes epistatic on *lic2A* that can account for the difference in the survival profiles between the *galE* and *lic2A* mutants. Specifically, this approach identifies secondary mutations that increase or decrease virulence phenotypes associated with deletion of *lic2A*. Our results implicated a diversity of genes in virulence when *lic2A* is deleted, including genes that influence the synthesis of cryptic LOS glycoforms, in addition to an unexpected genetic interaction in which deletion of *lic2A* specifically alleviated much of the requirement for *galE* in survival of NTHi in the lung. The results support a model in which an alternative LOS configuration controlled by precursor production levels modulates virulence properties of NTHi.

## Materials and Methods

### Media and *Haemophilus influenzae* Growth Conditions

The non-encapsulated Rd derivative of *H. influenzae* type d (RdAW) (Akerley et al., 2002), and nontypeable *H. influenzae* (NTHi) clinical isolates 375 (NTHi375) (Bouchet et al., 2003) and NT127 (Wong et al., 2013) were grown at 35°C ± 1.5°C and 5% CO_2_ in Brain Heart Infusion supplemented with 10 μg/ml NAD and 10 μg/ml hemin (sBHI) on agar plates or in sBHI broth. Strains were also grown in a chemically defined medium (MIc) or on MIc agar as described (Barcak et al., 1991). DNA was transformed into naturally competent *H. influenzae* as described (Barcak et al., 1991). Kanamycin (Km), gentamicin (Gm), erythromycin (Erm), tetracycline (Tet), 3,4-cyclohexenoesculetin-β-D-galactopyranoside (S-Gal), and D-xylose were added to sBHI at 20 μg/ml, 15 μg/ml, 10 μg/ml, 3 μg/ml, 300 μg/L, and 1 mM, respectively. S-Gal is a chromogenic substrate for β-galactosidase for detection of bacterial colonies with the *lac*^+^ phenotype; D-xylose is used for induction of the *lacZ* gene encoding β-galactosidase under the *xylA* promoter at the *xyl* locus (Wong et al., 2007). MIc agar contains sialic acid (SA) at 25 μg/ml supplemented (MIc^SA^) with or without 0.5 or 1% galactose.

### Plasmid and Strain Construction

Standard methods were used for strain and plasmid construction (Ausubel et al., 1995). Strains are listed in Table 1, and primer sequences in Table S1. Replacing the coding region of interest with the appropriate antibiotic resistance cassette via transformation with PCR products containing each gene knockout generated nonpolar deletion mutants in *H. influenzae*. Complementing strains were created by cloning the gene of interest for complementation into allelic exchange vector pXT10 for integration at *xyl* of the desired mutant recipients (Wong and Akerley, 2003). Detailed description of strain construction and other strains/plasmids is provided in Supplementary Text S1.

**Table 1:** *H. influenzae* strains and plasmids used in this study.

**Strains**	**Relevant features**	**Reference or source**
RdAW	Non-encapsulated *H. influenzae* Rd; referred to as Rd in this study	Akerley et al., 2002
NTHi375	Nontypeable *H. influenzae* clinical isolate 375	Bouchet et al., 2003
NT127	Nontypeable *H. influenzae* clinical isolate NT127	Wong et al., 2013
NT127siaB	NT127Δ*siaB*::*aphI;* Km^R^	This study
RdLacZ	Rd carrying pXELacZ2 at the *xyl* locus; *H. influenzae* expressing LacZ	This study
Δrep	Rd *licA*ΔCAAT; *licA* is phase on locked	Wong and Akerley, 2005
Δreplic2A	ΔrepΔ*lic2A*::*aacC1*; Gm^R^	This study
Rdlic2A	RdΔ*lic2A*::*aacC1*; Gm^R^	This study
RdgalE	RdΔ*galE*::*aphI*; Km^R^	Wong et al., 2013
Rdlic2AgalE	Rdlic2A Δ*galE*::*aphI*; Gm^R^ Km^R^; *lic2A galE* double deletion mutant	This study
RdgalK	RdΔ*galK*::*aacC1*; Gm^R^	This study
RdgalKgalE	RdgalK Δ*galE*::*aphI*; Gm^R^ Km^R^; *galK galE* double deletion mutant	This study
RdlsgE	RdΔ*lsgE*::*ermC'* expressed via the *trc* promoter; Erm^R^	This study
RdlsgD	RdΔ*lsgD*::*ermC'* expressed via the *trc* promoter; Erm^R^	This study
RdlsgC	RdΔ*lsgC*::*ermC'* expressed via the *trc* promoter; Erm^R^	This study
Rdlic2AlsgE	Rdlic2A Δ*lsgE*::*ermC'* expressed via the *trc* promoter; Gm^R^ Erm^R^	This study
	*lic2A lsgE* double deletion mutant	
Rdlic2AlsgD	RdΔlic2A Δ*lsgD*::*ermC'* expressed from the *trc* promoter; Gm^R^ Erm^R^	This study
	*lic2A lsgD* double deletion mutant	
Rdlic2AlsgC	RdΔlic2A Δ*lsgC*::*ermC'* expressed from the *trc* promoter; Gm^R^ Erm^R^	This study
	*lic2A* and *lsgC* double deletion mutant	
Rd^V^	Rd carrying empty vector pXT10 at the *xyl* locus; Tet^R^	Wong et al., 2007
Rdlic2A^V^	Rdlic2A carrying empty vector pXT10 at the *xyl* locus; Tet^R^	This study
RdgalE^V^	RdgalE carrying empty vector pXT10 at the *xyl* locus; Tet^R^	This study
RdgalElic2A^V^	RdgalElic2A carrying empty vector pXT10 at the *xyl* locus; Tet^R^	This study
RdgalE/galE^C^	RdgalE carrying *galE* expressed under its own promoter at the *xyl* locus; Km^R^ Tet^R^;	This study
	*galE* mutant containing *galE* complementing construct	
RdgalE lic2A/lic2${\text{A}}_{\text{ON}}^{\text{C}}$	RdgalElic2A carrying pLic2Aon at the *xyl* locus; Km^R^ Gm^R^ Tet^R^; *galE lic2A*	This study
	double deletion mutant complemented for *lic2A* mutation with *lic2A* phase-on locked	
375lic2A	NTHi375Δ*lic2A*::*aacC1*; Gm^R^	This study
375galE	NTHi375Δ*galE*::*aphI*; Km^R^	This study
375lic2AgalE	375galE Δ*lic2A*::*aacC1*; Km^R^ Gm^R^; *lic2A galE* double deletion mutant	This study
375siaB	NTHi375 carrying kanamycin resistance cassette inserted into *siaB*; Km^R^	Bouchet et al., 2003
**Plasmids**		
pXT10	Delivery vector for chromosomal expression at the *xyl* locus of *H. influenzae*,	Wong and Akerley, 2003
	contains *tetAR* tetracycline resistance cassette; Tet^R^; referred to as 'V'	
pXELacZ2	pXT10 carrying *lacZ* expressed from the *xylA* promoter	Rosadini et al., 2011
pFLOB4300	Vector carrying *ermC*'-*rpsL_*gc*_* cassette containing erythromycin selectable marker	Johnston and Cannon, 1999
pTrcHIs2B	Source of *E. coli trc* promoter	ThermoFisher
pGalE	pXT10 carrying Rd *galE* expressed from its own endogenous promoter	This study
pLic2Aon	pXT10 carrying Rd *lic2A*(ΔCAAT) expressed from its own endogenous promoter	This study

### Absorption of Sera

Absorption of sera was performed as described (Loeb, 1984) with modifications (Supplementary Text S1), using strains RdgalElic2A/lic2${\text{A}}_{\text{on}}^{\text{C}}$ and RdgalElic2A grown on MIc agar containing sialic acid (25 μg/ml) (MIc^SA^).

### Western Blotting

Bacteria grown on MIc^SA^ overnight were resuspended at ~0.4 OD_600_ units in Hank's Balanced Salt Solution with calcium and magnesium chloride (HBSS++) and NuPAGE LDS Sample buffer containing lithium dodecyl sulfate (ThermoFisher). Whole-cell lysates treated with or without 1 mg/ml proteinase K were separated on Invitrogen NuPAGE 4–12% Bis-Tris Protein Gels (ThermoFisher) using 2-(N-morpholino) ethanesulfonic acid (MES) running buffer, pH 7, transferred to PVDF membranes (Millipore) and blocked with TBS-0.1% Tween 20 containing 1% dry milk for 30 min at 24°C. Membranes were incubated with serum pre-absorbed with strains RdgalElic2A/lic2${\text{A}}_{\text{on}}^{\text{C}}$ (GalE^−^, Lic2A^+^) or RdgalElic2A^V^ (GalE^−^, Lic2A^−^) diluted 1:120 in TBS-0.1% Tween 20 for 17 h at 4°C on a rotary shaker. Membrane-bound IgM and IgG were detected using alkaline phosphatase-conjugated anti-human IgM and IgG (Sigma) at a 1:1,000 dilution in TBS-0.1% Tween 20 and 5-bromo-4-chloro-3-indolyl phosphate/nitro blue tetrazolium (BCIP/NPT) purple liquid substrate system (Sigma). Coomassie Blue staining of undigested samples was performed with a Colloidal Blue Staining Kit (ThermoFisher). Size markers were SeeBlue Plus2 pre-stained protein standards.

### Serum Bactericidal Assays

Bacteria were harvested after overnight growth on MIc^SA^ with or without 0.5 or 1% galactose and resuspended in HBSS++ for serum bactericidal assays as previously described (Wong et al., 2011), with minor modifications (Supplementary Text S1). In designated experiments, 10 mM EGTA and 10 mM magnesium chloride (MgCl_2_) were added to NHS to selectively block the classical and lectin, but not the alternative complement pathway (Platts-Mills and Ishizaka, 1974; Des Prez et al., 1975). Bactericidal assays using strain specific absorbed sera were performed as described above except IgG/IgM antibody depleted human complement (HC) pooled serum (Pel-Freez Biologicals) was used as a source of active complement. Percent survival is calculated as indicated in the figure legends.

### Flow Cytometry

Rd and NTHi 375 strains were grown and harvested as described for the serum bactericidal assays above. Binding of serum antibodies to bacteria incubated with either NHS^Δ*i*^ or absorbed NHS^Δ*i*^ was detected with anti-human IgM and IgG conjugated to fluorescein isothiocyanate (FITC) (SouthernBiotech) on a NovoCyte flow cytometer with analysis using NovoExpress software (ACEA Biosciences, Inc., San Diego, CA). The reported median fluorescence intensities (MFI) of bacterial populations in replicate samples were obtained after subtraction of the background FITC-conjugated antibody only fluorescence values obtained for each strain.

### Biofilm Assay

Biofilm formation was assessed by the microtiter plate assay (O'toole, 2011). NTHi 375 strains grown to log phase in sBHI were diluted to OD_600_ of 0.01 in sBHI and seeded at 200 μl per well in replicates in polystyrene 96-well flat bottom microtiter plates (Corning Incorporated, Kennebunk, ME) in the presence of 0.1% galactose, 25 μg/ml sialic acid, 0.1% *N*-acetylglucosamine or in combinations of the three reagents for 24 hr at 36°C with 5% CO_2_. After assessing growth by measuring optical density at 600 nm, cultures were decanted, and biofilms quantified by crystal violet staining detected as absorbance at 595 nm, as previously described (O'toole, 2011).

### *H. influenzae* Colonization and Selection of Transposon Mutant Libraries in the Murine Lung Model

Single strain colonization and *in vivo* competition assays were essentially as described previously (Gawronski et al., 2009; Wong et al., 2013; Supplementary Text S1). This model measures survival of *H. influenzae* in the otherwise healthy mammalian lung, resulting in transient weight loss and morbidity with clearance by approximately day 4. Attenuated mutants in this model are more rapidly eliminated, indicating loss of potential virulence genes. For *in vivo* selection of the *H. influenzae* Rd transposon insertion libraries in strains Δrep (parent) and Δreplic2A (isogenic *lic2A* deletion mutant), genomic DNA representing 10^5^ mutants (Gawronski et al., 2009) was used to transform these two strains to Km^R^ or Km^R^Gm^R^ to generate mutant banks termed parent library and *lic2A* library, respectively. We used the Δrep strain containing *licA* that is phase-on locked for LOS PC epitope to prevent phase variation at this locus, which has been shown previously to influence phenotypes of *lic2A* mutants (Langereis and Weiser, 2014). Forty microliter containing 10^7^ CFU of each transposon insertion library was inoculated into the nares of five mice per strain. The remaining portion of the inoculum was processed for genomic DNA representing the input libraries. Output libraries were recovered from lungs at ~24 h post-infection, grown on sBHI agar plates and collected for genomic DNA isolation for transposon insertion mapping analysis. Experiments were conducted with approval and in accordance with guidelines of the Institutional Animal Care and Use Committee at the University of Mississippi Medical Center (Jackson, MS).

### Sequencing of Transposon Junctions and Analysis of Sequence Data

Genomic DNA from parent and *lic2A* libraries was isolated pre- and post-selection and sequenced using true single molecule DNA sequencing (tSMS) technology developed by SeqLL (Woburn, MA), formerly Helicos BioSciences (Cambridge, MA) (Thompson et al., 2012). The tSMS reads were aligned to the *H. influenzae* Rd KW20 genome sequence, GenBank: L42023.1 (National Center for Biotechnology Information, NCBI) using HeliSphere software (SeqLL) with the “best-only” setting and other parameters taken as defaults, assigned to genes using a revised version of NCBI protein table NC_000907 and filtered to remove reads corresponding to more than one location on the genome as described previously (Wong et al., 2013). The *mariner* transposon *Himar1* inserts specifically into TA dinucleotides in target DNA (Lampe et al., 1996; Rubin et al., 1999). In the input parent and *lic2A* mutant banks, 64,348 (~50%) and 55,028 (~42%) of the 131,955 total possible chromosomal TA target sites were found to have sustained unique insertions, respectively. The number of sequencing reads mapping to the genome resulted in averages of 27-fold and 12-fold coverage of each TA insertion site within the parent and *lic2A* mutant libraries, respectively. Of the 1,724 predicted or annotated genes in the genome, we excluded 443 (from parent library) and 481 (from *lic2A* mutant library) loci essential for growth *in vitro* from our analysis as well as duplicated genes, genes with ≤ to 5 reads and those with only one TA insertion within the first 95% of their coding regions. Additional detailed analyses including classification of genes based on their fitness profiles, assigning gene essentiality and threshold requirements for genes considered to contribute to fitness during lung infection is provided in Supplementary Text S1.

## Results

### Genome-Wide Genetic Interaction Screen

A possibility to account for the difference in survival phenotypes of *lic2A* and *galE* mutants in the lung model would be the presence of other genes that have functions redundant with *lic2A*. In order to identify gene(s) in this category, we used HITS to examine the fitness of a large bank of *mariner* transposon insertion mutants (~75,000) in a *lic2A* deletion background vs. the control parental strain pre- and post-selection in the mouse lung model. This approach can unmask gene(s) that become required in the absence of *lic2A* during infection, potentially accounting for the difference in the survival profiles between the *galE* and *lic2A* mutants we observed previously. Moreover, the *lic2A* gene is subject to reversible inactivation by phase variation mediated by 5′-CAAT-3′ repeats in its coding region (High et al., 1996), and this approach can reveal genes that may be required during natural infection in which *lic2A* can reversibly switch off by this mechanism.

**Figure 2:**
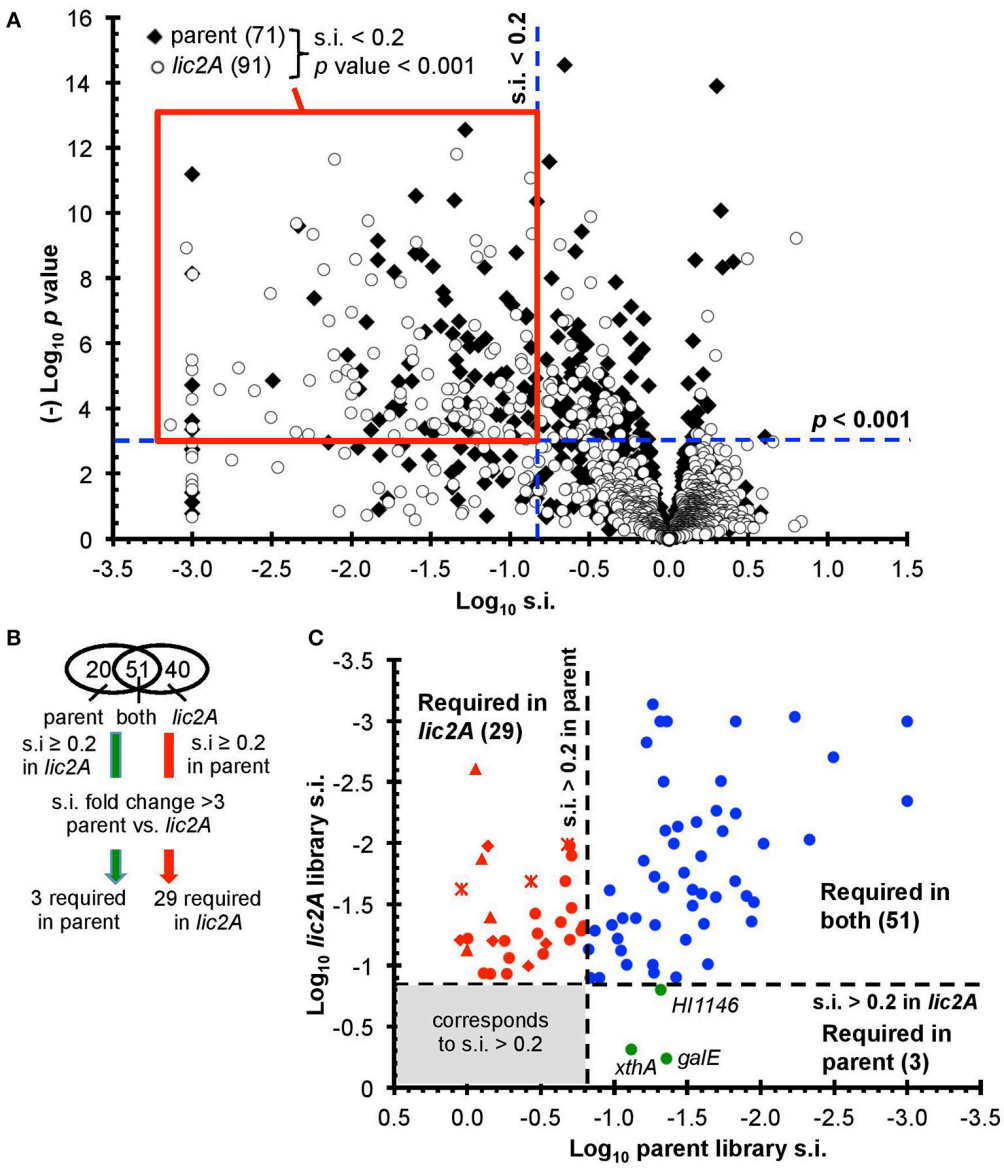
Genome-wide genetic interaction screen via HITS evaluating fitness of *H. influenzae* mutants in a *lic2A* deletion background in a mouse lung model. **(A)** Mutant fitness (survival index, s.i.) of candidate virulence genes in parent and *lic2A* deletion with no observed effects on fitness *in vitro* was plotted against significance (*p*-value). Red box demarcates the genes of interest meeting the requirements for further analysis (s.i. <0.2, *p*-value <0.001). **(B)** Venn diagram displaying these genes of interest sorted into categories. Further criteria were imposed to identify candidate genes required for infection in parent, *lic2A* only or in both. **(C)** Fitness of the transposon mutants attenuated in the parent or *lic2A* or in both was plotted against each other, highlighting their differential s.i. phenotypes. The 3 genes required for parent alone (green) or 29 in *lic2A* alone (red) are displayed (*lsg*, crosses; *lic1*, triangles; *yrb*, diamonds) (Table 2). The 51 genes required in both (blue) *in vivo* conditions are listed in Table S4.

After excluding duplicated genes and genes with few insertions including those that are essential *in vitro*, the remaining 1,236 and 1,197 genes in the parent and *lic2A* mutant libraries, respectively, were further analyzed for roles in fitness in the mouse lung model (Figure 2A) (Materials and Methods and Supplementary Text S1). Fitness is expressed as a survival index (s.i.) representing the ratio of insertions detected after infection (output) to those detected in the *in vitro* grown inoculum (input). Values of s.i. <0.2 and *p* < 0.001 were set as requirements for genes to be considered to contribute to fitness during infection, resulting in 71 genes identified as required for infection in the parent, and 91 in the *lic2A* mutant bank dataset, of which 51 were required in both. A further cutoff of a fold difference of s.i. >3 between the two mutant banks was set as the criterion for a gene to be considered required for survival in one library but not the other, resulting in 3 and 29 genes that are considered uniquely required in the lung model by either the parent or *lic2A* mutant, respectively (Figures 2B,C). S.i. values for *galE* (0.04) and *lic2A* (0.8) in the parent library were consistent with our previous s.i. results for *galE* (0.025, 0.03) and *lic2A* (0.4, 1) in the mouse lung model (Gawronski et al., 2009; Wong et al., 2013). HITS data for all protein coding genes are listed in Table S2 and *in vitro* essential genes identified using the EL-ARTIST pipeline (Pritchard et al., 2014) are listed in Table S3. As expected, and further validating the method, 90% of genes within the common core set of 51 required for both parent and in the *lic2A* mutant background (Table S4) were found in our previous HITS results for *H. influenzae* infection in the murine lung model and have been discussed previously (Gawronski et al., 2009; Wong et al., 2013).

Importantly, deletion of *lic2A* changed the genes required in the lung and yielded two categories of interest: mutations that resulted in survival defects only in the *lic2A* mutant background, and mutations that enhanced survival in this mutant but caused attenuation in the parent (Table 2). Several genes that were required only in the *lic2A* deletion background have roles in influencing the cell envelope integrity or changes in LOS structure: e.g., *dacA* encoding penicillin-binding protein 5 involved in peptidoglycan biosynthesis (Sauvage et al., 2008), the *lic1* locus (*licABCD*) required for phosphorylcholine modification of the LOS in *H. influenzae* (Weiser et al., 1997), the *lsgCDE* genes involved in carbohydrate extension of the cryptic LOS structures (Hood et al., 2004b; Johansen et al., 2010) and the *vacJ*/*yrbCDEF* genes, encoding an ABC transport system that functions in maintenance of outer membrane lipid asymmetry (Malinverni and Silhavy, 2009) and influences the spatial arrangement of LOS in NTHi (Nakamura et al., 2011). Other genes in this category had annotated functions in DNA recombination/repair (*recG, xerC*), DNA recombination with regulatory roles (*fis, ihfA*), protein turnover/translation (*lon, deaD*), energy metabolism (*aspA, pgi*), L-tryptophan biosynthesis (*trpG*), transport of phosphate (*pstA*) and iron (*hitC*), and regulatory roles (*sspA, phoB*). Three genes were required in the parental strain background, but not required in the *lic2A* deletion background, indicating an unexpected suppression of virulence defects by the *lic2A* mutation. These genes have known or proposed functions in DNA repair **(***xthA*), RNA degradation (*HI1146*, putative *rapZ*-like homolog) and LOS biosynthesis (*galE*).

**Table 2:** Genes required in *H. influenzae* parent or *lic2A* library.

**Genes required in parent library**						
**Gene ID**	**Gene**	**Product/predicted function**	**Parent library**		***lic2A*** **library**	
			**s.i.^a^**	***p*** **value**	**s.i.^a^**	***p*** **value**
HI0041	*xthA*	Exonuclease III	0.08	1.3E-05	0.5	3.4E-01
HI0351	*galE*	UDP-glucose 4-epimerase	0.04	5.2E-07	0.6	8.2E-05
HI1146	*rapZ-*like	Putative RNase adaptor protein	0.05	2.2E-07	0.2	2.5E-04
**Genes required in** ***lic2A*** **library**						
HI0029	*dacA*	Penicillin-binding protein 5	0.2	6.2E-07	0.06	7.1E-10
HI0099	*hitC*	Iron(III) ABC transporter ATP-binding protein	0.8	2.3E-01	0.12	8.2E-05
HI0231	*deaD*	ATP-dependent RNA helicase	0.5	4.4E-02	0.12	3.2E-06
HI0462	*lon*	ATP-dependent proteinase	0.2	1.5E-07	0.01	2.6E-09
HI0534	*aspA*	Aspartate ammonia-lyase	0.5	4.3E-04	0.09	1.5E-05
HI0676	*xerC*	Site-specific tyrosine recombinase	0.6	1.5E-01	0.06	3.6E-04
HI0718	*vacJ (mlaA)*	Lipoprotein	1.1	8.8E-01	0.06	8.8E-04
HI0980	*fis*	Factor for inversion stimulation DNA binding protein	0.3	2.1E-04	0.05	3.0E-04
HI1084	*yrbC (mlaC)*	ABC transporter; substrate-binding protein	0.7	1.8E-01	0.06	3.5E-04
HI1085	*yrbD (mlaD)*	ABC transporter periplasmic protein	0.4	1.0E-03	0.10	6.8E-04
HI1086	*yrbE (mlaE)*	ABC transporter permease	0.7	5.0E-01	0.01	2.3E-05
HI1087	*yrbF (mlaF)*	ABC transporter ATPase	0.3	2.7E-04	0.07	6.6E-05
HI1159m	*-*	Thioredoxin domain-containing protein	0.2	1.0E-02	0.05	2.3E-04
HI1313	*ihfA*	Integration host factor subunit alpha	1.0	9.8E-01	0.06	8.5E-05
HI1379	*phoB*	Phosphate regulon transcriptional regulatory protein	0.2	1.5E-04	0.04	1.8E-04
HI1381	*pstA*	Phosphate ABC transporter permease	0.2	3.4E-04	0.05	6.7E-05
HI1388	*trpG*	Anthranilate synthase component II	0.3	8.3E-04	0.04	4.3E-04
HI1441	*sspA*	Stringent starvation protein A	0.2	3.3E-03	0.02	3.3E-05
HI1537	*licA*	lic-1 operon protein, choline kinase	0.9	2.1E-01	0.002	2.9E-05
HI1538	*licB*	lic-1 operon protein, choline transporter	1.0	9.5E-01	0.07	1.6E-09
HI1539	*licC*	lic-1 operon protein, pyrophosphorylase	0.8	1.0E-02	0.01	1.1E-08
HI1540	*licD*	lic-1 operon protein, choline transferase	0.7	1.3E-03	0.04	2.5E-05
HI1576	*pgi*	Glucose-6-phosphate isomerase	0.3	1.1E-06	0.08	1.4E-06
HI1654	*-*	Hypothetical protein	0.7	9.4E-02	0.12	4.7E-05
HI1658	*yraP*	Predicted outer membrane lipoprotein	0.2	8.3E-05	0.01	1.6E-04
HI1696	*lsgE*	Glycosyltransferase/*N*-acetylglucosaminyltransferase	0.2	1.2E-04	0.01	8.7E-06
HI1697	*lsgD*	Galactosyltransferase	1.1	7.8E-01	0.02	3.2E-04
HI1698	*lsgC*	Putative galactosyltransferase	0.4	9.6E-04	0.02	1.3E-08
HI1740	*recG*	ATP-dependent DNA helicase	0.2	1.2E-05	0.03	2.9E-04

a *survival index*.

### Evaluation of *lsg* Interaction With *lic2A*

**Figure 3:**
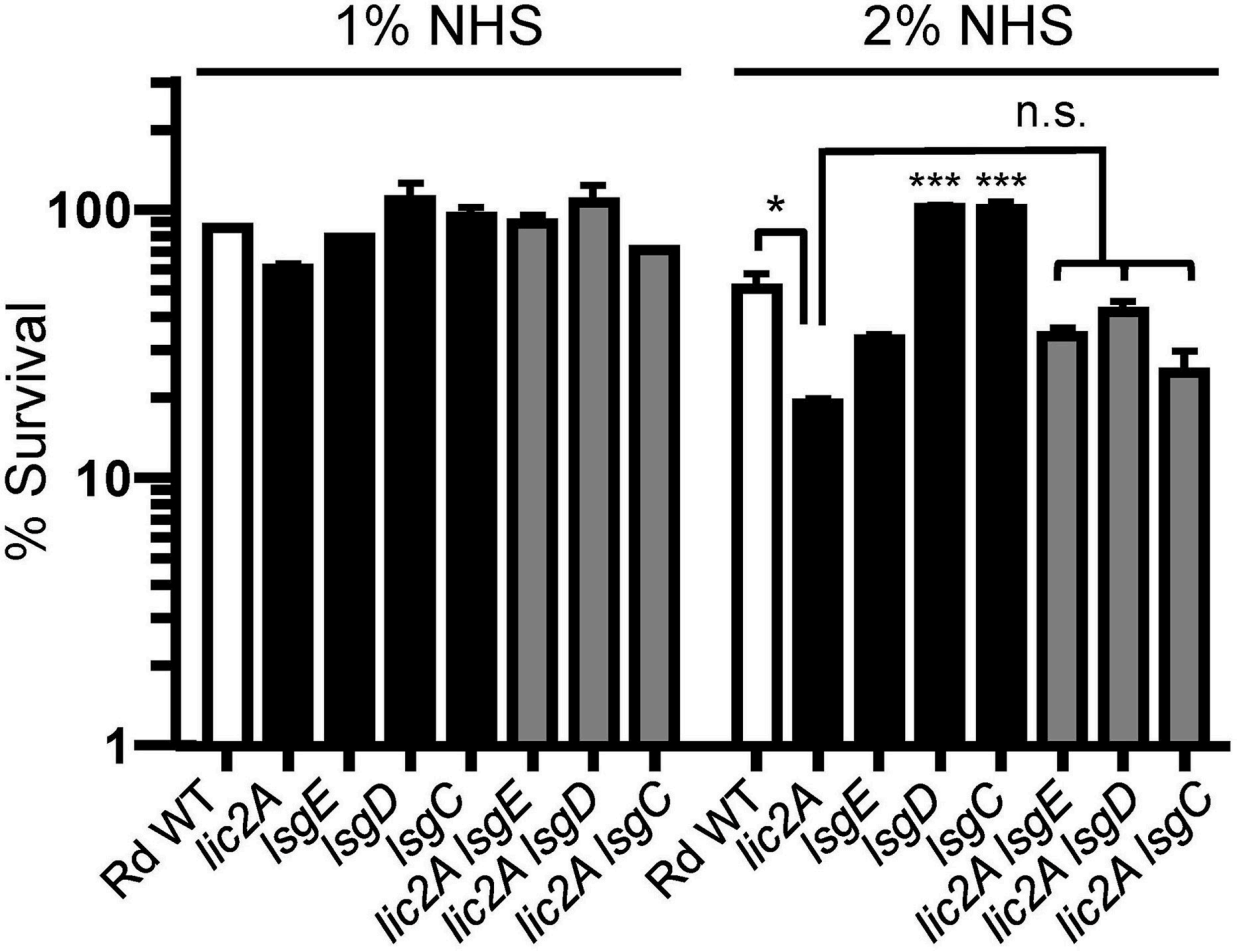
Deletion of *lsg* genes in parent or the *lic2A* mutant backgrounds does not significantly increase serum sensitivity. Viability of Rd wild-type (WT) and isogenic deletion mutants *lic2A, lsgE, lsgD, lsgC*, and double deletion mutants *lic2A lsgE, lic2A lsgD*, and *lic2A lsgC* grown in sBHI medium was assayed following incubation with 1 and 2% normal human serum (NHS) at 37°C for 30 min. Percent survival is the ratio of CFU recovered from serum treated samples at 30 min to CFU recovered from heat inactivated serum (NHS^Δ*I*^) treated samples. The mean of two independent samples is shown. The *lsgD* and *lsgC* mutants each exhibited statistically significant increase in survival compared to all other samples at 2% NHS. Statistical significance is evaluated by one-way ANOVA with Bonferroni's multiple comparison test (***p < 0.001; *p < 0.05).

Several genes of the *lsg* locus encoding glycosyltransferases were implicated in our genetic screen, and may have redundant functions with *lic2A* that could be responsible for suppressing *in vivo* defects of *lic2A* mutants. The HITS data indicate that mutations in these *lsg* genes in the *lic2A* mutant background gave rise to low survival indices suggesting their requirement in the absence of *lic2A*. As complement evasion is an important defense mechanism of *H. influenza*e, bactericidal assays with normal human serum (NHS) were used to evaluate whether these genes confer complement resistance in this context. Results of the serum bactericidal assays with the double *lsg lic2A* mutants showed that the *lsgE, lsgD*, and *lsgC* deletions in the *lic2A* mutant background do not increase serum bactericidal killing at statistically significant levels compared to the *lic2A* single mutant (Figure 3) indicating that the apparent attenuation of the double *lic2A lsg* mutants *in vivo* is likely effected by host defense mechanism(s) other than complement. Alternatively, lack of significant differential serum killing between the *lic2A* mutant and *lsg lic2A* double mutants could also result from a lack (or low levels) of expression or activity of the *lsg* gene products *in vitro*. These genes will require further studies to fully evaluate their interactions with *lic2A*.

### Deletion of *lic2A* Increases Serum Resistance of the *galE* Mutant

**Figure 4:**
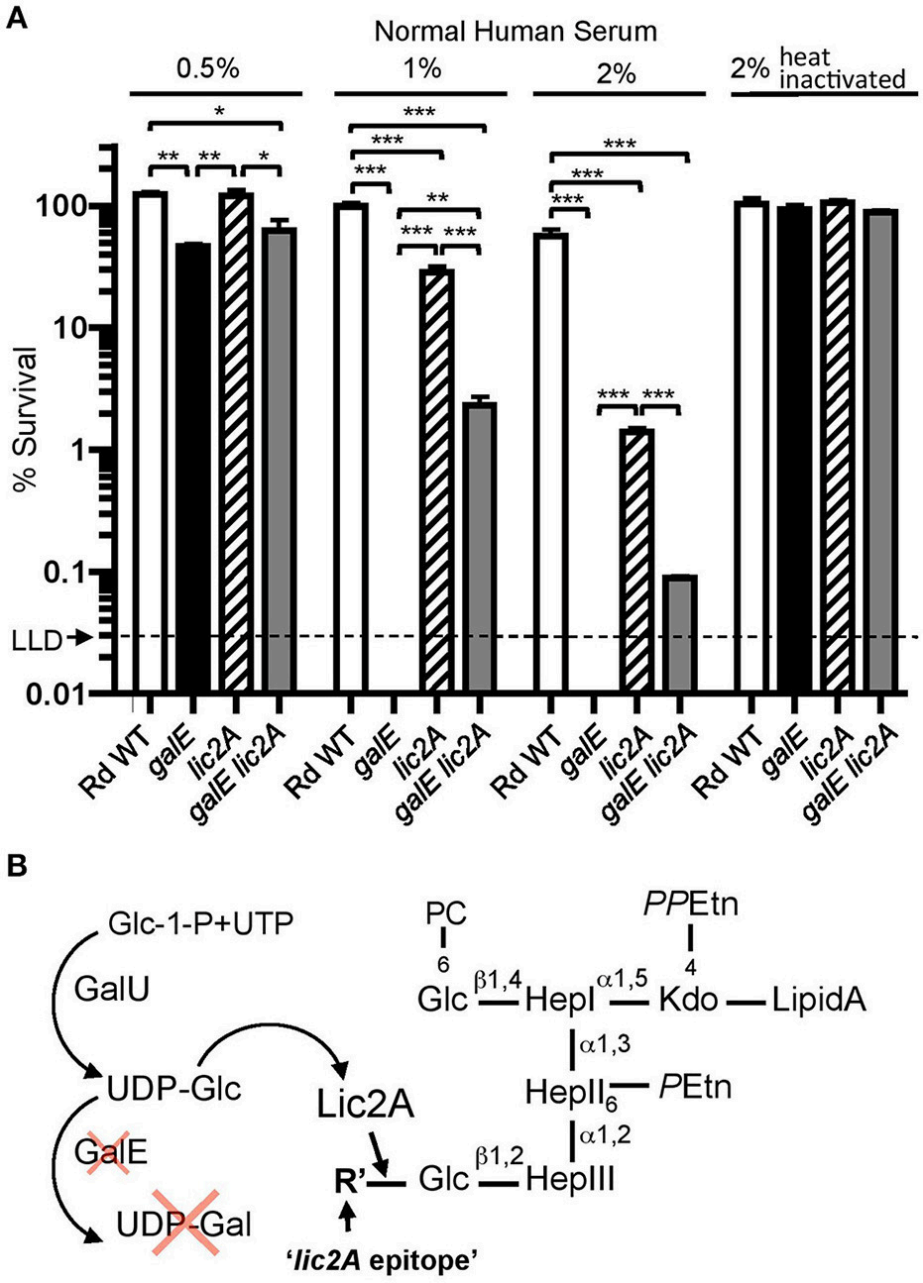
Deletion of *lic2A* increases serum resistance of the *galE* mutant. **(A)** Survival of Rd wild-type (WT) and isogenic single deletion mutants *galE, lic2A* and double deletion mutant *galE lic2A* grown in sBHI medium was assayed following incubation with 0.5, 1, and 2% NHS at 37°C for 30 min. Percent survival is the ratio of CFU recovered from samples treated with either NHS or NHS^Δ*i*^ at 30 min to CFU recovered from untreated samples. Log-transformed survival ratios were evaluated by one-way ANOVA with Bonferroni's multiple comparison test (*p < 0.05; **p < 0.01; ***p < 0.001). The mean of triplicate samples is shown. LLD, lower limit of detection. **(B)** Proposed model to account for complement sensitivity of the *galE* mutant resulting from addition of a *lic2A* dependent alternative epitope in the absence of galactose. GalU, glucosephosphate uridylyltransferase, R' is inferred to be Glc (see Results and Discussion).

Unexpectedly, our genetic screen revealed that the *galE* mutant was restored to virulence in the *lic2A* mutant background. To investigate the mechanism of this restoration, we performed bactericidal assays with NHS to determine the sensitivity profile of Rd wild-type compared to isogenic deletion mutants of *galE, lic2A*, and a *galE lic2A* double deletion mutant following growth in sBHI. Results showed that the *galE* mutant is more sensitive than the *lic2A* mutant to killing at 0.5, 1, and 2% NHS. Interestingly, relative to the *galE* mutant, the *galE lic2A* double deletion mutant exhibited significantly increased resistance to serum killing (Figure 4A). A hypothesis to account for serum sensitivity of the *galE* mutant is the proposed addition of an alternative structure to the LOS by Lic2A in the absence of UDP-Gal (Figure 4B). That this alternative *lic2A* dependent epitope is likely a glucose residue is supported by previous structural studies indicating the presence of an unexpected glucose residue at this position in an *H. influenzae galE* mutant (Masoud et al., 2008) (further described in Discussion).

Serum bactericidal assays typically have used *H. influenzae* grown in sBHI, an undefined rich medium. It has been noted that this medium contains trace amounts of sialic acid that could increase serum resistance in *H. influenzae* via sialylation of the LOS (Hood et al., 1999). Similarly, we found that wild-type NTHi strain NT127 was ~8-fold more resistant to 2% NHS compared to an isogenic *siaB* mutant following growth in sBHI in the absence of supplemental sialic acid (Figure S1). The *siaB* gene encodes CMP-Neu5Ac synthetase, which catalyzes the conversion of sialic acid to CMP-Neu5Ac, the activated nucleotide sugar donor essential for LOS sialylation. Therefore, these results indicate that wild-type NTHi are able to scavenge sialic acid from sBHI for LOS modification. To further refine our analysis of the bactericidal effect of NHS on the *galE, lic2A*, and *galE lic2A* mutants, we used a chemically defined medium, MIc, to control the levels of sialic acid in our assays, exploiting the fact that *H. influenzae* lacks the complete biosynthetic pathway for sialic acid synthesis and must acquire it from exogenous sources (Fleischmann et al., 1995; Vimr et al., 2000; Severi et al., 2007).

**Figure 5:**
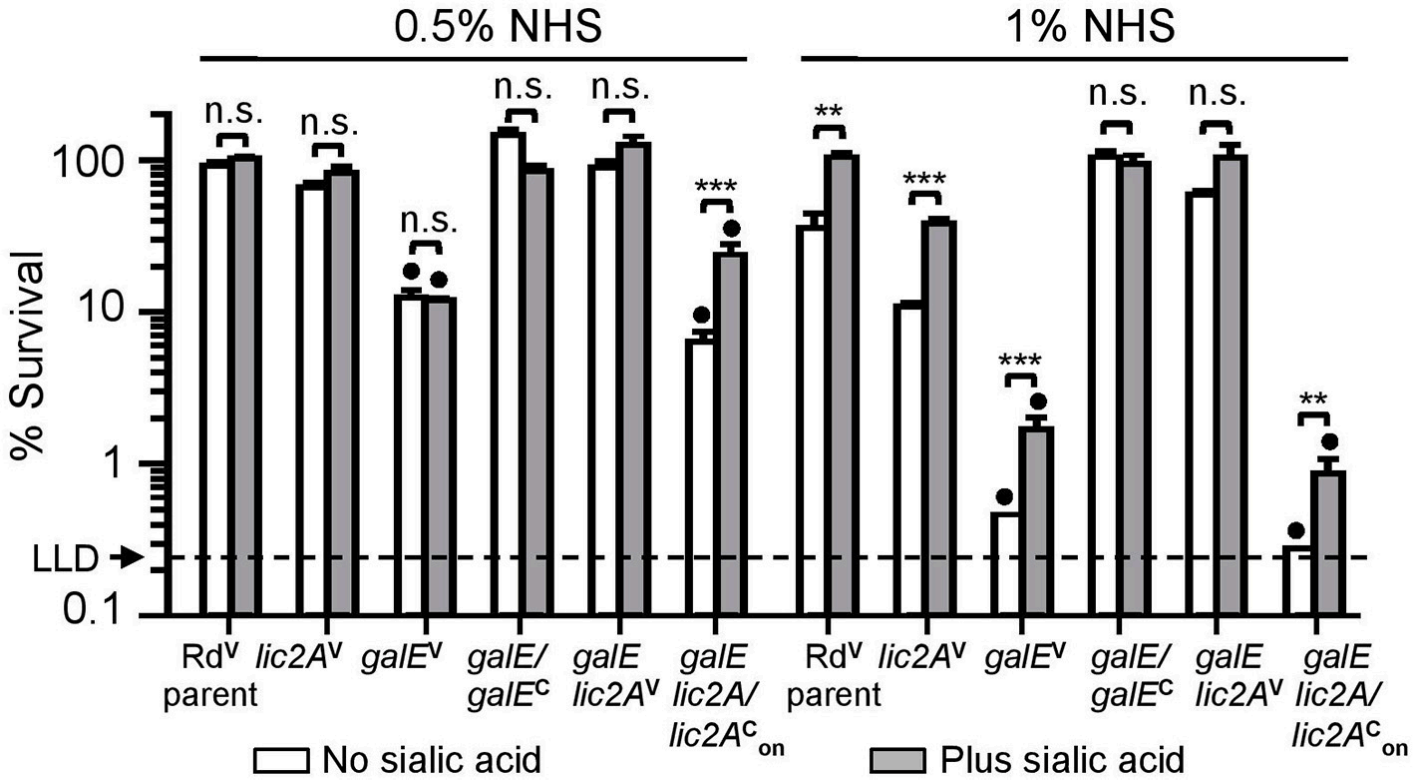
Deletion of *lic2A* increases serum resistance of the *galE* mutant when grown in the absence or presence of sialic acid on MIc medium. Viability of Rd containing empty cloning vector (V) (Rd^V^parent), *lic2A* mutant containing V (*lic2A*^V^), *galE* mutant containing V (*galE*^V^), *galE* mutant with complementing copy of *galE in trans* (*galE*/*galE*^C^), *galE lic2A* mutant containing V (*galE lic2A*^V^), *galE lic2A* mutant with complementing copy of phase-on locked *lic2A in trans*, (*galE lic2A*/*lic2*$A_{\text{on}}^{\text{C}}$) following incubation with 0.5% and 1% NHS at 37°C for 30 min. Percent survival is the ratio of CFU recovered at 30 min from serum treated samples to CFU recovered from samples treated with NHS^Δ*i*^. Statistical significance of log-transformed survival ratios between each strain ± sialic acid is indicated (one-way ANOVA with Bonferroni's multiple comparison test; (**p < 0.01; ***p < 0.001; n.s., not statistically significant). *galE*^V^ and *galE lic2A*/*lic2*$A_{\text{on}}^{\text{C}}$ mutants significantly differed (denoted with black circles, *p* < 0.001) from all other strains except each other in −/+ sialic acid in both NHS concentration. The mean of triplicate samples is shown. LLD, lower limit of detection is approximately 0.25% survival.

Serum bactericidal assays with Rd and mutants grown on MIc agar in either the presence or absence of supplemental sialic acid prior to treatment with 0.5 and 1% NHS confirmed sensitivity of the *galE* mutant to NHS killing (Figure 5). Because *lic2A* is subject to phase variation, we created strain RdgalE lic2A/lic2${\text{A}}_{\text{on}}^{\text{C}}$, a *galE lic2A* double deletion mutant containing *lic2A* complemented with a copy of *lic2A* that has been phase-on locked by deletion of CAAT repeats (Supplementary Text S1). This strain displayed a serum sensitivity phenotype comparable to that of the *galE* mutant, consistent with sequencing data indicating that our *galE* mutant is phase-on for *lic2A* (Supplementary Text S1). As expected, complementation of *galE* rescued serum resistance, and deletion of *lic2A* in the *galE* mutant background was also confirmed to rescue serum resistance similar to the results seen in Figure 4A. Because sialic acid is abundant on mucosal surfaces, we grew *H. influenzae* in MIc containing sialic acid, MIc^SA^ for subsequent serum bactericidal assays and IgM binding studies to more closely resemble the host environment.

### Exogenous Galactose Alleviates Serum Sensitivity of the *galE* Mutant

**Figure 6:**
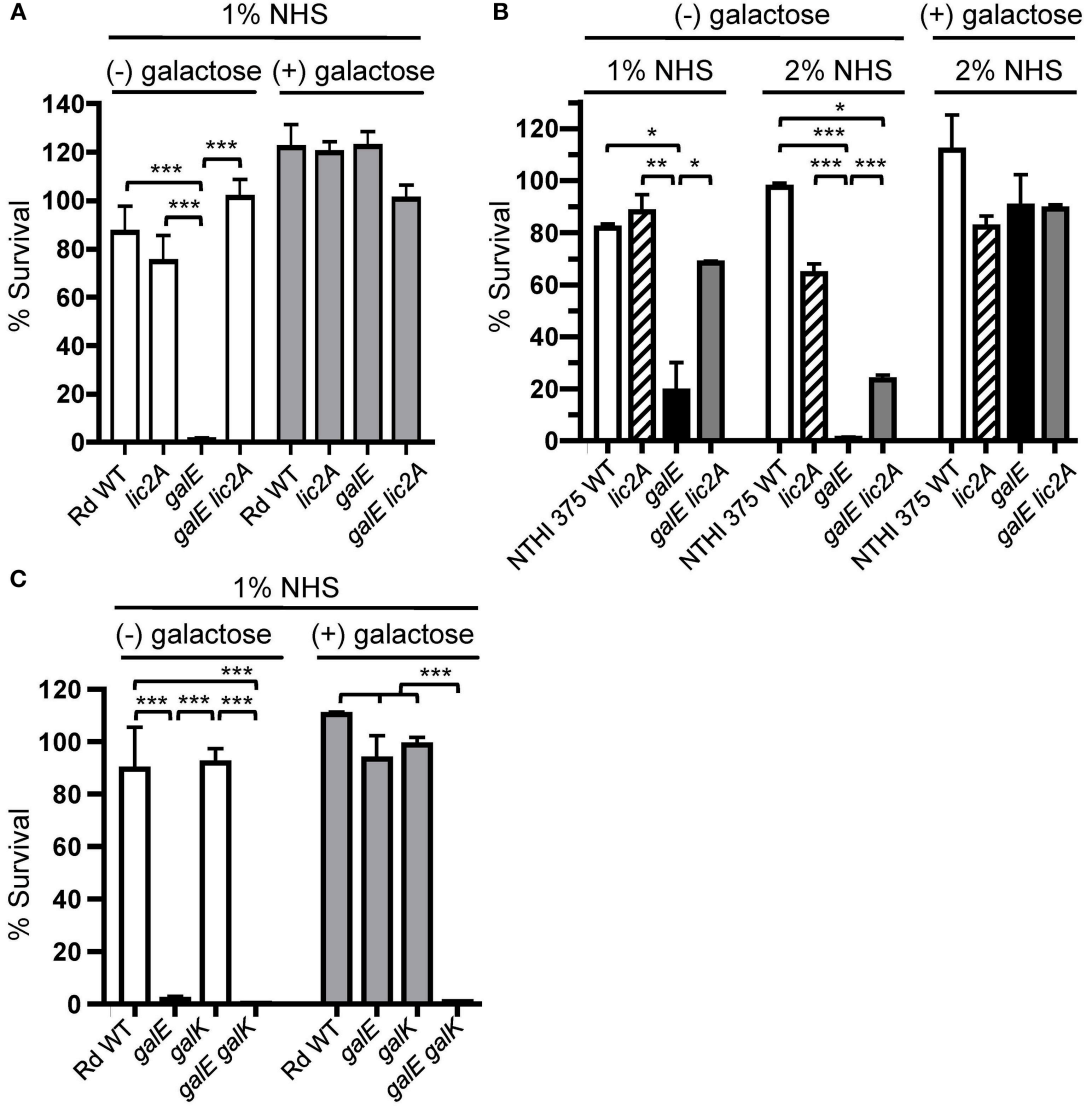
Deletion of *lic2A* or growth on galactose alleviates sensitivity of the *galE* mutant to bactericidal effects of NHS and blocking galactose catabolism in the *galE* mutant confers serum sensitivity. Viability of *H. influenzae* wild-type (WT) Rd **(A,C)** and NTHi strain 375 **(B)** and isogenic single and double deletion mutants in their respective strain backgrounds grown on MIc medium containing sialic acid (MIc^SA^) in the absence (-) or presence (+) of 1 or 0.5% galactose for Rd and NTHi 375, respectively, following incubation with the percent of NHS indicated at 37°C for 30 min. Percent survival is the ratio of CFU recovered at 30 min from serum treated samples to CFU recovered from NHS^Δ*i*^ treated samples. Log-transformed survival ratios were evaluated by one-way ANOVA with Bonferroni's multiple comparison test (*p < 0.05, **p < 0.01,***p < 0.001). The mean of duplicate assays is shown. Lower limit of detection is approximately 0.25% survival.

The g*alE* mutant is unable to generate UDP-Gal via the endogenous Leloir pathway, in which UDP-glucose pyrophosphorylase converts Glc-1-P to UDP-Glc, which the GalE epimerase then converts to UDP-Gal (Frey, 1996). If defects in LOS and serum resistance are caused by a deficiency in UDP-Gal, then resistance should be restored by provision of the *galE* mutant with an exogenous source of galactose, which can be converted to UDP-Gal via the exogenous Leloir pathway. The exogenous Leloir pathway requires *galK*, encoding galactokinase, which produces the phosphorylated intermediate galactose-1-phosphate, which is then converted to UDP-Gal by galactose-1-phosphate uridylyltransferase encoded by *galT* (Frey, 1996). Results of serum bactericidal assays showed that exogenous galactose restored serum resistance to *galE* mutants in both Rd and NTHi 375 strain backgrounds. A slight growth inhibition of the *galE* mutants by galactose under these conditions was observed (data not shown), however the rescued serum resistance of the *galE* mutants by galactose overcame this defect. The *galE* mutants of both of these strains grown in the absence of exogenous galactose were sensitive to NHS, and deletion of *lic2A* in their respective *galE* mutant backgrounds restored a significant level of resistance (Figures 6A,B).

If catabolism of exogenous galactose is blocked in the *galE* mutant by deletion of *galK*, thus depriving cells of a source of activated galactose precursor for *lic2A*, then expression of the alternative *lic2A* structure is predicted to result in serum sensitivity. Serum bactericidal assays with the *galE galK* double deletion mutant confirmed that the exogenous galactose utilization pathway is required for serum resistance of the *galE* mutant (Figure 6C). Both *galE* and *galE galK* mutants were sensitive to serum killing when grown in the absence of galactose. As expected, galactose alleviated the serum sensitivity of the *galE* mutant, and the *galK* deletion mutant does not exhibit serum sensitivity regardless of exogenous galactose. However, the *galE galK* double mutant remained sensitive to serum killing even when supplied with exogenous galactose, confirming that blocking exogenous galactose catabolism in the *galE* mutant confers serum sensitivity.

### Increased IgM Binding in Rd and NTHi 375 Correlates With Increased Serum Sensitivity

**Figure 7:**
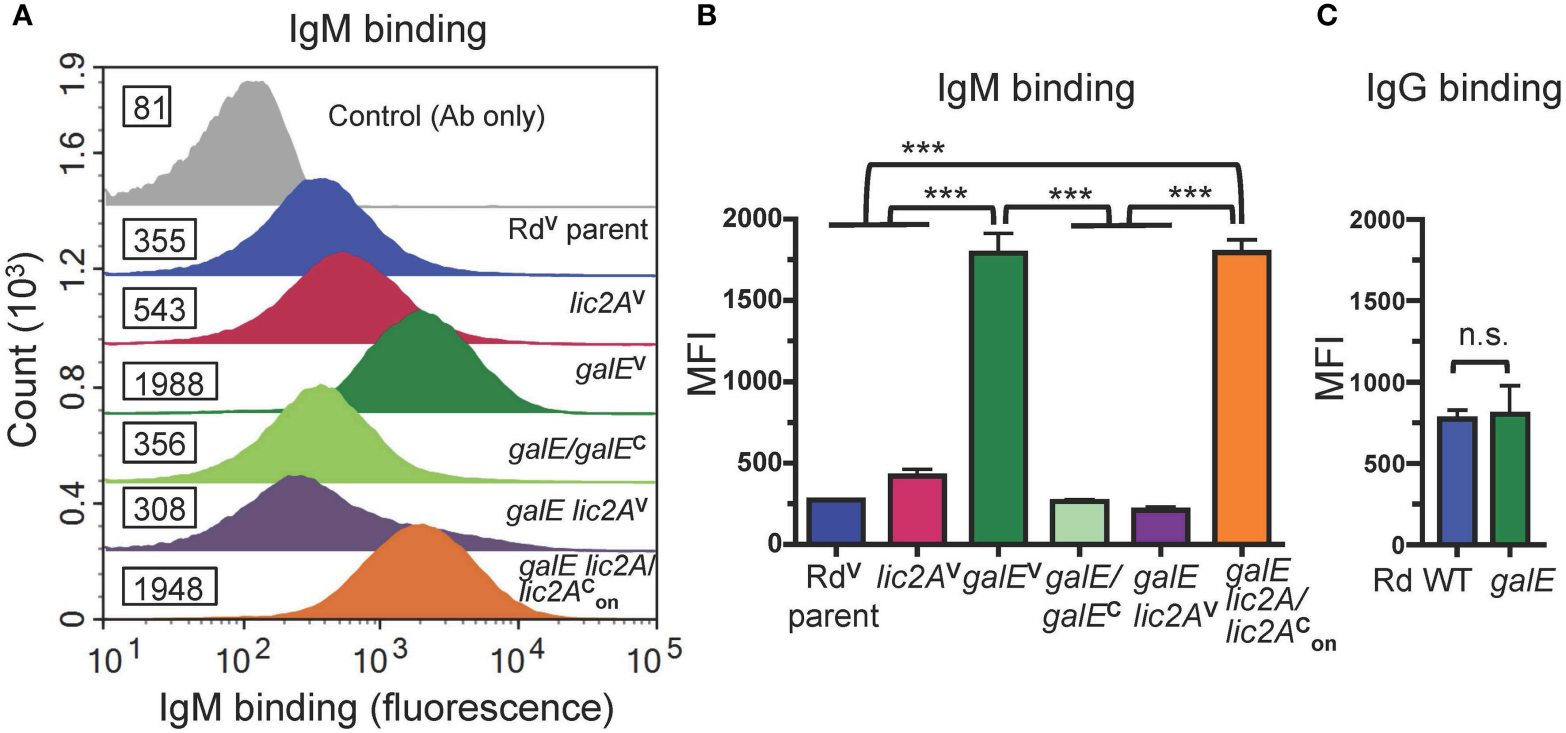
Increased binding of IgM but not IgG to the *galE* mutant and *galE lic2A* containing *lic2A* phase-on locked. **(A)** Rd strains grown in MIc^SA^ were incubated with NHS^Δ*i*^ at 20% final concentration at 37°C for 30 min followed by detection via flow cytometry with anti-human IgM conjugated to FITC. X-axis, fluorescence; Y-axis, counts. Numbers alongside histograms indicate the median fluorescence intensity (MFI) of the bacterial population. Control antibody (Ab) only fluorescence values are similar for all strains and one representative is shown (Rd^V^ parent). One representative experiment of two reproducible repeats is shown. **(B)** IgM and **(C)** IgG binding for the mean of replicate samples of which one is shown in **(A)** for IgM. Statistical significance was evaluated by one-way ANOVA with Bonferroni's multiple comparison test (***p < 0.001; n.s., not statistically significant).

**Figure 8:**
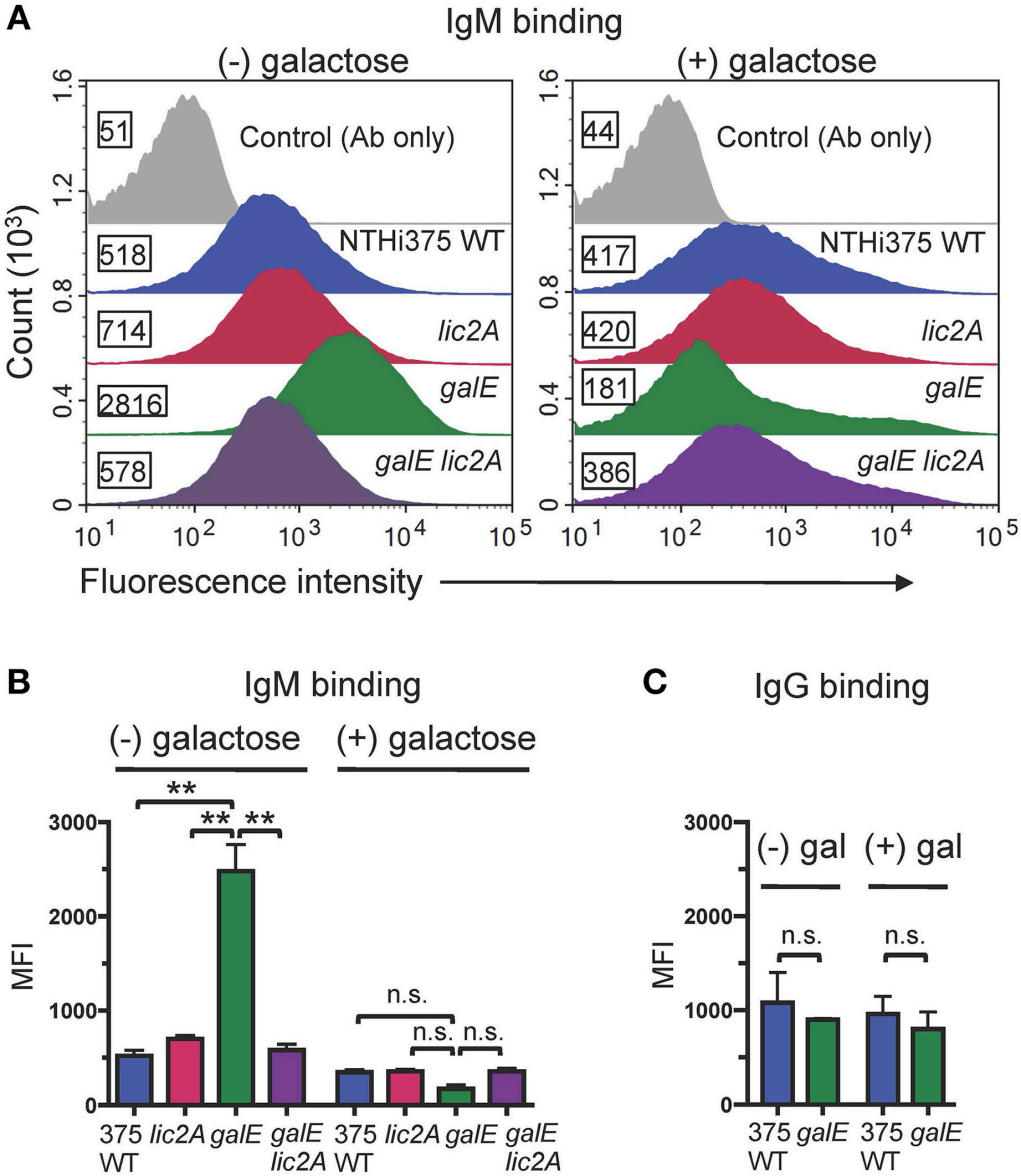
Increased binding of IgM but not IgG to the NTHi 375 *galE* mutant. **(A)** IgM binding to NTHi 375 wild-type (WT) and isogenic mutants grown in the absence or presence of 0.5% galactose in MIc^SA^ then incubated with 20% NHS^Δ*i*^ followed by detection with anti-human IgM conjugated to FITC via flow cytometry. X-axis, fluorescence; Y-axis, counts. Numbers alongside the histograms indicate the MFI of the bacterial population. Control Ab only fluorescence values are similar for all strains and only one representative is shown (NTHi 375 WT). One representative experiment of two reproducible repeats is shown. **(B)** IgM and **(C)** IgG binding for the mean of replicate samples of which one is shown in **(A)** for IgM. Statistical significance was evaluated by one-way ANOVA with Bonferroni's multiple comparison test (**p < 0.01; n.s., not statistically significant).

Antibodies, including IgM and to a lesser extent IgG, are potent activators of the classical complement pathway (CP), which is required for maximal bactericidal activity of complement against NTHi (Williams et al., 2001; Wong et al., 2011; Rosadini et al., 2014). Therefore, we investigated whether a functional *lic2A* gene in the *galE* mutant confers increased antibody binding leading to serum sensitivity. Increased IgM antibody binding of the *galE* mutant containing *lic2A* phase-on locked and the Rd *galE* mutant (also phase-on for *lic2A*) (Figures 7A,B) correlated with their serum sensitivity when grown without exogenous galactose (Figures 5, 6A). Also consistent with serum sensitivity results, deletion of *lic2A* in the *galE* mutant background and complementation of the *galE* mutant both reduced IgM binding to the levels seen in wild type. No differential IgG binding between Rd wild-type and the *galE* mutant was detected (Figure 7C). As expected, no significant differential binding of IgM was detected between Rd wild-type and the *galE* mutant when grown in the presence of galactose (Figure S2). Similarly, increased IgM binding of the NTHi 375 *galE* mutant (Figures 8A,B) correlated with increased serum sensitivity of this *galE* mutant grown in the absence, but not in the presence of exogenous galactose and deletion of *lic2A* in the *galE* mutant abrogated this increased binding correlating with decreased serum sensitivity (Figure 6B). As with Rd, there was no statistically significant differential binding of IgG between NTHi 375 wild-type and its isogenic *galE* mutant grown under either condition (Figure 8C).

### Deletion of *lic2A* in the *galE* Mutant Background Improves Survival in the Lung

**Figure 9:**
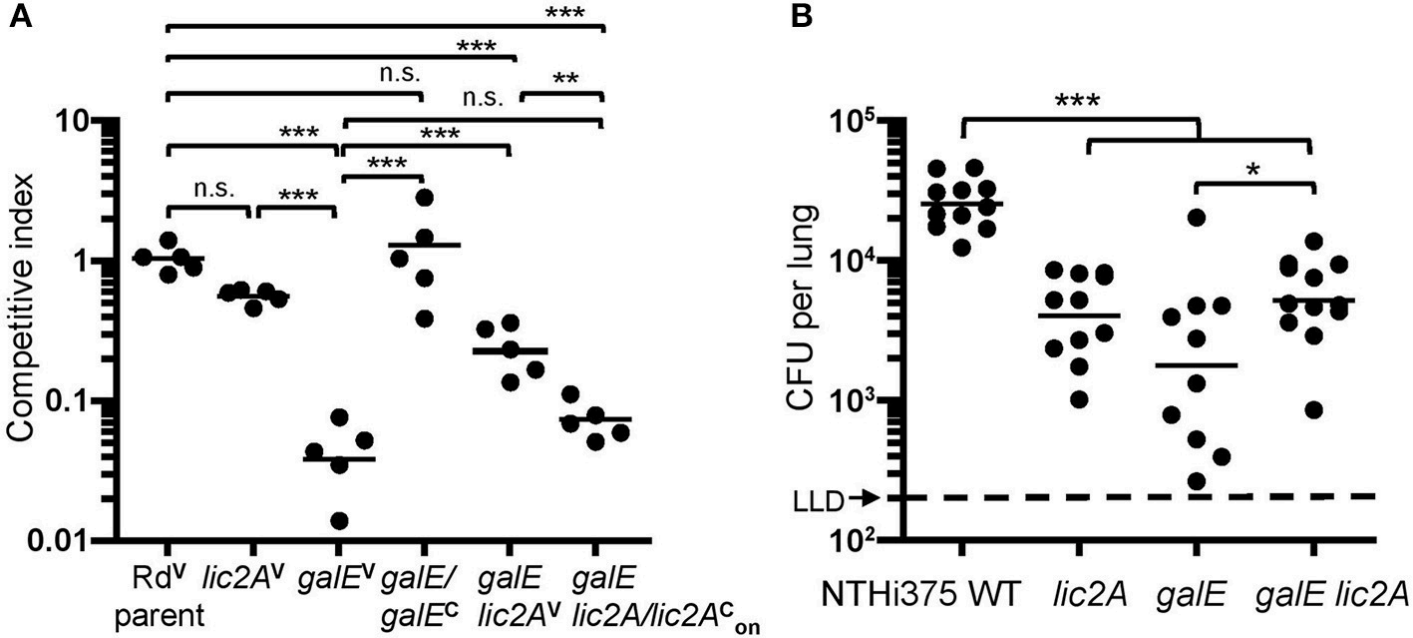
Deletion of *lic2A* in the *galE* mutant background partially rescues attenuation of the *galE* mutant for lung colonization in both Rd and NTHi strains. *H. Influenzae* Rd or NTHi 375 and isogenic mutants in their respective background strains recovered from lungs of C57BL/6 mice 24 h after intranasal inoculation. **(A)** *H. influenzae* Rd strains as described in Table 1 were co-inoculated with an equal amount of an *H. influenzae* reference strain expressing *lacZ* (RdLacZ) and recovered from mice (*n* = 5). Ratio of CFU of the experimental strains (LacZ–) to competitor strain (LacZ+) is reported as the competitive index (CI). Bars represent the geometric mean ratio of CFU of each designated strain relative to the LacZ+ reference strain. CI fold differences of parent vs. each of the mutants: *lic2A*^*V*^ (1.9x), *galE*^*V*^ (24x), *galE lic2A*^*V*^ (4.3x), and *galE lic2A*/*lic2*$A_{\text{on}}^{\text{C}}$ (14x) and between *galE lic2A*^*V*^ *vs*. each of the mutants: *galE*^*V*^ (5.5x), *galE lic2A*/*lic2*$A_{\text{on}}^{\text{C}}$ (3.3x). **(B)** NTHi 375 wild-type (WT) and isogenic deletion mutants recovered from mice (*n* = 10–12). Bars represent the geometric mean CFU per lung. LLD, lower limit of detection. Fold differences in mean CFU recovered for WT vs. each of the mutants: *lic2A* (5.5x), *galE* (6.9x), and *galE lic2A* (4.3x) and between *galE* vs. *galE lic2A* (1.6x). Differences were evaluated via one-way ANOVA with Bonferroni's multiple comparison test of log-transformed values (*p < 0.05; **p < 0.01; ***p < 0.001; n.s., not statistically significant).

To confirm the *in vivo* survival phenotype of *galE* mutants in the *lic2A* mutant background as identified in HITS data, survival of Rd, NTHi 375 and their respective isogenic mutants *(lic2A, galE, galE lic2A*) was evaluated in the mouse lung model. Supporting the HITS data, *galE* mutants exhibited the most severe attenuation in survival relative to wild-type strains in both backgrounds. The NTHi 375 *lic2A* mutant was moderately attenuated while the Rd *lic2A* mutant had no significant defect. Deletion of *lic2A* in the *galE* mutants of Rd and NTHi 375 partially rescued the persistence defect of the *galE* mutants in both strain backgrounds (Figures 9A,B). Moreover, locking *lic2A* in the phase-on state in the Rd *galE* mutant (*galE lic2A/lic2*$A_{ON}^{c}$) decreased persistence similar to that seen with the *galE* mutant, and complementation (*galE/galE*^*c*^) restored survival of the *galE* mutant in the lung (Figure 9A).

### The Classical Pathway of Complement Is Required for Differential Serum Killing of the *galE* Mutant vs. Wild-Type NTHi 375

Increased IgM binding to the *galE* mutants (GalE^−^, Lic2A^+^) (Figures 7, 8) implicated the classical pathway of complement in their increased sensitivity to serum killing relative to the parental strains. To evaluate the role of the CP in differential killing, we performed bactericidal assays with NHS containing EGTA and MgCl_2_, which selectively blocks the classical and lectin pathways but not the alternative pathway. Chelation of calcium by EGTA blocks the calcium ion dependent assembly of the C1 complex of the classical pathway (Lepow et al., 1963). Calcium ions are also required for binding by C-type animal lectins (Iobst and Drickamer, 1994) and for activation of the lectin pathway by mannose-associated serine proteases (Wallis and Dodd, 2000).

**Figure 10:**
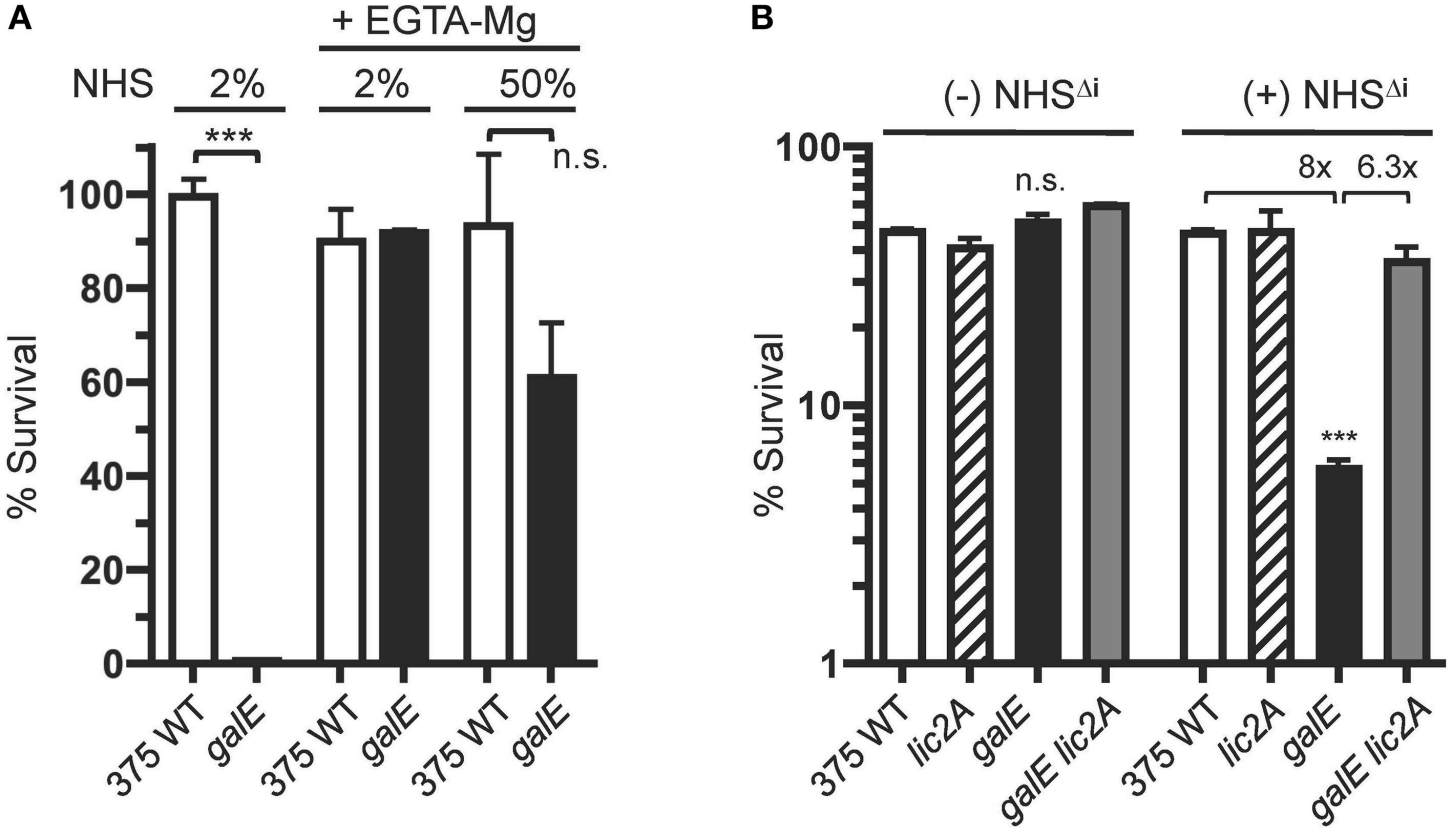
Killing of the NTHi 375 *galE* mutant by complement requires serum antibody. **(A)** Survival of NTHi 375 wild-type (WT) and isogenic *galE* mutant grown on MIc^SA^ medium assayed following incubation with the indicated percentage of NHS in the absence or presence of EGTA and MgCl_2_ at 37°C for 30 min. Percent survival is the ratio of CFU recovered from serum treated samples (±EGTA and MgCl_2_) at 30 min to CFU recovered from NHS^Δ*i*^ treated samples (±EGTA and MgCl_2_). **(B)** Antibody-dependent killing of the *galE* mutant by NHS. Viability of NTHi 375 wild-type (WT) and isogenic deletion mutants grown on MIc^SA^ following incubation with or without 10% unabsorbed NHS^Δ*i*^ followed by incubation with 3% IgG/IgM antibody depleted human complement active serum (HC) at 37°C for 30 min. Percent survival is the ratio of the number of CFU recovered at 30 min. relative to the input CFU at *T* = 0 min. of incubation. Log transformed survival ratios were evaluated by one-way ANOVA with Bonferroni's multiple comparison test (***p < 0.001; n.s., not statistically significant). In **(B)**, the *galE* mutant differed significantly from the other three strains within the NHS^Δ*i*^ treatment group. The mean of duplicate samples is shown. Lower limit of detection is ~0.25% survival.

Results in Figure 10A show that the NTHi 375 *galE* mutant was highly sensitive to 2% NHS as expected, but its resistance to killing is equivalent to wild type in 2% NHS with EGTA-MgCl_2_. Moreover, in NHS with EGTA-MgCl_2_ at 50%, the *galE* mutant approaches the survival levels of the wild type, retaining ~60% survival. We note, however, that at 50% EGTA-MgCl_2_, some killing of the *galE* mutant may occur by the alternative pathway, however this difference was not statistically significant. That the differential killing in NHS requires antibody mediated activation of the classical pathway was confirmed in bactericidal assays with NHS depleted of IgM and IgG as a source of active human complement (HC), with or without supplementation with heat inactivated NHS (NHS^Δ*i*^) as a source of antibody (Figure 10B). Without a source of antibody from NHS^Δ*i*^, there was no differential killing between strains, however in the presence of serum antibody, the *galE* mutant was killed 8-fold more compared to wild type, and deletion of *lic2A* in the *galE* mutant restored resistance to wild-type levels.

### Bactericidal Activity Against the *galE* Mutant Requires Recognition of a *lic2A*-Dependent LOS Epitope

**Figure 11:**
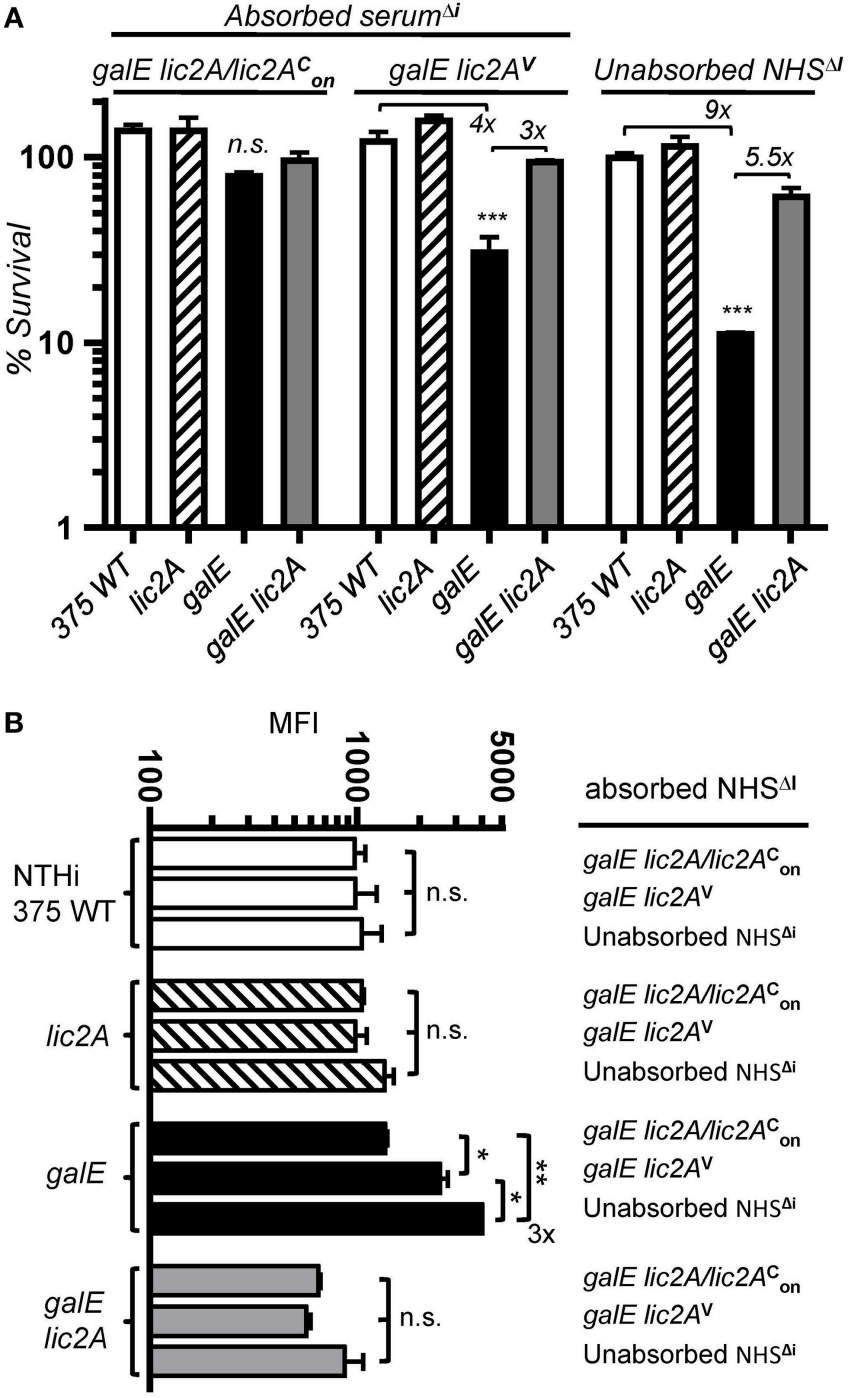
Absorption experiments indicating a *lic2A* dependent epitope in the *galE* mutant that confers increased killing by serum and IgM binding. **(A)** Serum pre-absorbed of the antibody that targets the *lic2A* dependent epitope in the *galE* mutant promotes decreased killing of the *galE* mutant. Viability of the indicated strains grown on MIc^SA^ following incubation with 10% NHS^Δ*i*^ pre-absorbed with strains RdgalElic2A/lic2${\text{A}}_{\text{on}}^{\text{C}}$ (GalE^−^, Lic2A^+^) or RdgalElic2A^V^ (GalE^−^, Lic2A^−^) vs. control unabsorbed NHS^Δ*i*^, followed by incubation with 3% HC at 37°C for 30 min. Percent survival is the ratio of CFU recovered from absorbed or unabsorbed serum treated samples after HC incubation to CFU recovered from non-serum treated (buffer only) samples after HC incubation. Lower limit of detection is approximately 0.25% survival. Differences between the *galE* mutant vs. the other three strains within a treatment group were statistically significant by ANOVA, ***p < 0.001. **(B)** Serum pre-absorbed of the antibody targeting the *lic2A* dependent epitope in the *galE* mutant contains decreased IgM specific to the *galE* mutant. Strains grown on MIc^SA^ were incubated with 10% NHS^Δ*I*^ pre-absorbed with RdgalElic2A/lic2${\text{A}}_{\text{on}}^{\text{C}}$ or RdgalElic2A^V^ or control unabsorbed NHS^Δ*I*^ at 37°C for 30 min followed by detection via flow cytometry with anti-human IgM conjugated to FITC. IgM binding from the mean of replicate samples. Log-transformed survival ratios and MFI values were evaluated by one-way ANOVA with Bonferroni's multiple comparison test (*p < 0.05; **p < 0.01; n.s., not statistically significant).

To determine whether antibody dependent killing results from recognition of an epitope unique to the *galE* mutant, we performed bactericidal assays in HC supplemented with NHS^Δ*i*^ depleted of specific antibody by absorption. Sera were absorbed with a *galE* strain RdgalElic2A/lic2${\text{A}}_{\text{on}}^{\text{C}}$ (GalE^−^, Lic2A^+^) expressing *lic2A* locked phase-on or a *galE lic2A* double deletion strain RdgalElic2A^V^ (GalE^−^, Lic2A^−^) predicted to lack the epitope. NTHi 375 and isogenic *lic2A, galE* and *galE lic2A* mutants were evaluated in serum killing assays with HC supplemented with depleted sera or control unabsorbed NHS^Δ*I*^ (Figure 11A). Results showed that the *galE* mutant was less sensitive to serum killing when treated with the serum pre-absorbed with strain RdgalElic2A/lic2${\text{A}}_{\text{on}}^{\text{C}}$. In contrast, sera pre-absorbed with strain RdgalElic2A^V^ or control unabsorbed NHS^Δ*i*^ retained increased bactericidal activity against the *galE* mutant relative to wild type, *lic2A* and *galE lic2A* strains. Similar results were obtained with the same set of mutants in the Rd background (Figure S3). The decrease in serum sensitivity of the *galE* mutant treated with RdgalElic2A/lic2${\text{A}}_{\text{on}}^{\text{C}}$ pre-absorbed serum correlated with a parallel decrease in IgM binding by the *galE* mutant treated with this absorbed serum relative to unabsorbed serum or serum absorbed with the *lic2A* deficient strain, RdgalElic2A^V^ (Figure 11B).

**Figure 12:**
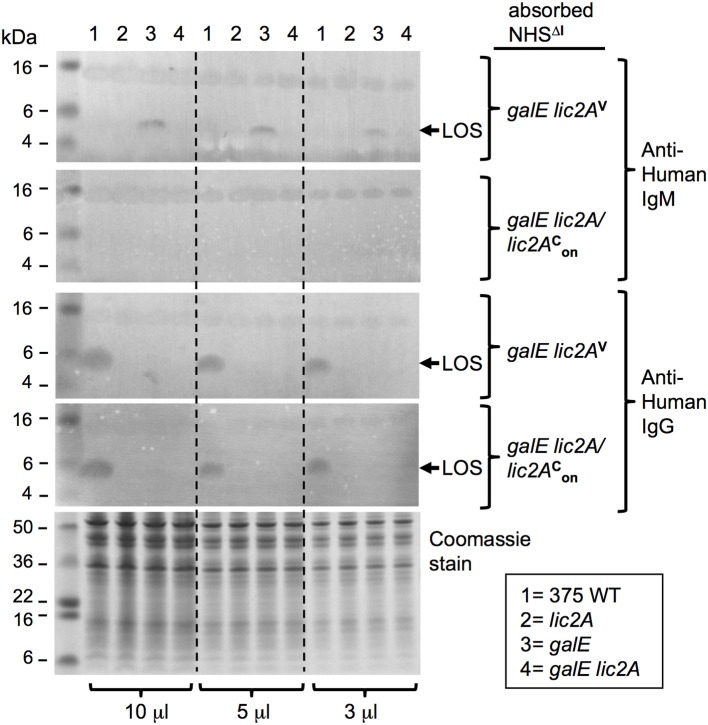
IgM binding to a *lic2A* dependent LOS epitope in the *galE* mutant. Whole-cell lysates from NTHi 375 and the indicated isogenic mutants grown on MIc^SA^ were digested with proteinase K and separated by NuPAGE 4–12% Bis-Tris gels followed by western immunoblotting. Membranes were incubated with serum pre-absorbed with strains RdgalElic2A/lic2${\text{A}}_{\text{on}}^{\text{C}}$ (GalE^−^, Lic2A^+^) or RdgalElic2A^V^ (GalE^−^, Lic2A^−^) followed by incubation with alkaline phosphatase conjugated anti-human IgM or IgG. Equal sample concentration was verified by Coomassie Blue staining of undigested samples. Each sample was electrophoresed at three different concentrations. Arrows indicate ~5kDa LOS; pre-stained protein standards in left lane.

Additional evidence for a *lic2A* dependent epitope recognized by IgM on the LOS of the *galE* mutant was obtained with immunoblots containing protease treated bacterial lysates probed with the two absorbed sera (Figure 12). IgM in the serum absorbed with the *galE lic2A* double deletion mutant bound to an ~5 kDa band, the expected size of the LOS. This IgM binding band was present in the *galE* mutant lysate, but was completely absent in lysates of NTHi 375 wild-type, *lic2A*, and *galE lic2A* deletion strains. A 15 kDa band, likely representing a protease resistant protein, bound equal amounts of IgM in all strains with both absorbed sera. Reactivity of IgM with the ~5 kDa band was completely eliminated with serum absorbed with the *galE* deletion strain. Consistent with a role for IgM but not IgG in complement sensitivity of the *galE* mutant, IgG binding to the *galE* mutant was not increased relative to other strains, but rather was undetectable in each mutant strain, while wild type exhibited substantial IgG reactivity with both absorbed sera despite its greater degree of complement resistance.

### Decreased UDP-Galactose Production and Increased Exogenous UDP-GlcNAc Precursor Availability Stimulate Biofilm Formation

**Figure 13:**
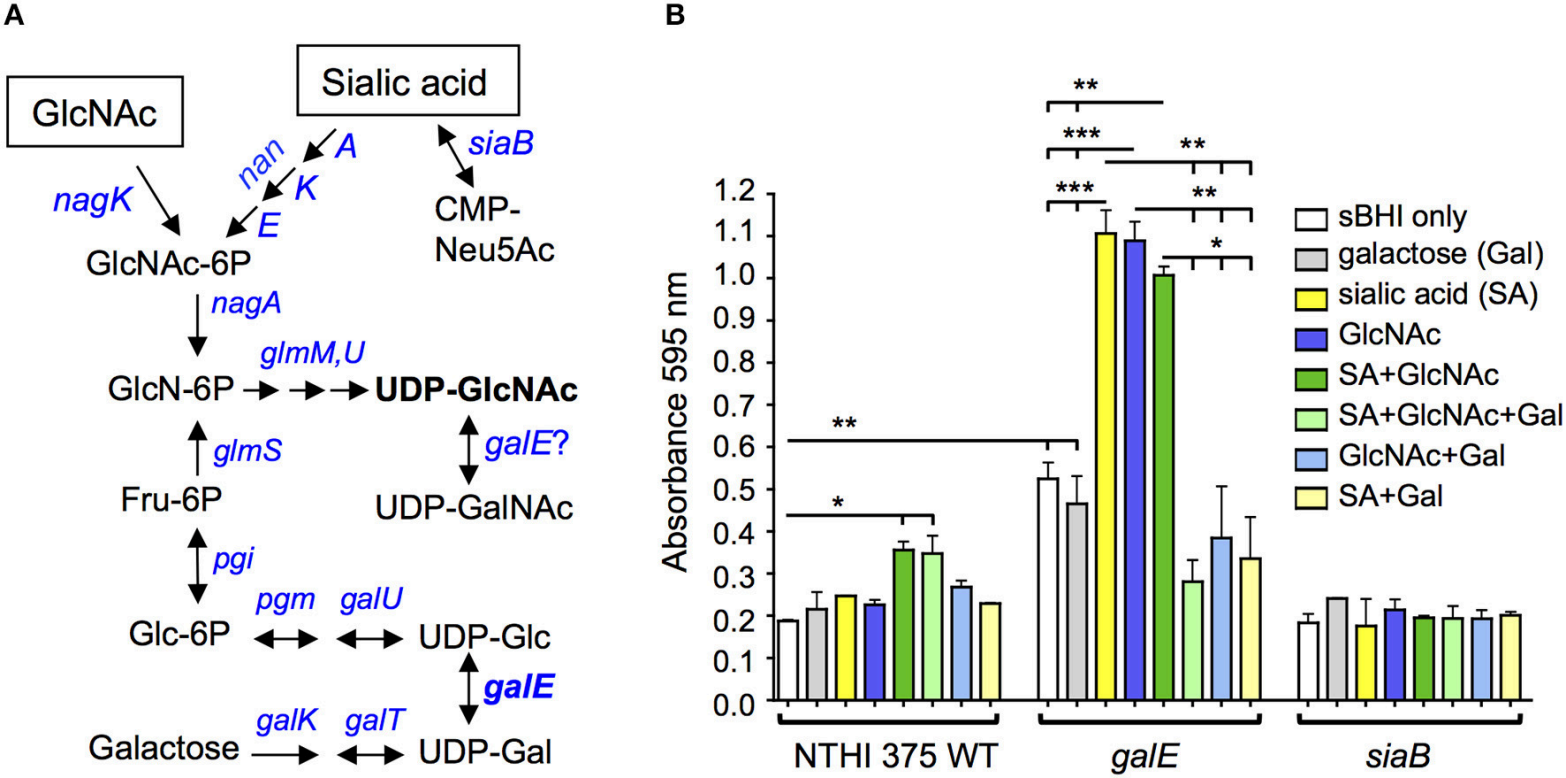
Exogenous UDP-GlcNAc precursors fuel biofilm production. **(A)** Amino sugar and nucleotide sugar metabolic pathway for generation of UDP-GlcNAc in *H. influenzae* (adapted from KEGG (Kyoto Encyclopedia of Genes and Genomes) pathway database (https://www.genome.jp/kegg/pathway.html). **(B)** NTHi 375 wild-type (WT) with isogenic mutants *galE* and *siaB* were grown in sBHI for 24 hr in the presence of galactose, sialic acid, *N*-acetylglucosamine (GlcNAc) or combinations of the three in microtiter plates and biofilm formation was quantified by crystal violet staining. Log-transformed values were evaluated by one-way ANOVA with Bonferroni's multiple comparison test (*p < 0.05; **p < 0.01; ***p < 0.001). GlcNAc-6P, *N*-acetylglucosamine 6-phosphate; GlcN-6P, D-glucosamine 6-phosphate; UDP-GlcNAc, UDP-*N*-acetylglucosamine; UDP-GalNAc, UDP-*N*-acetylgalactosamine; Fru-6P, β-D-fructose 6-phosphate; Glc-6P, α-D-glucose 6-phosphate; CMP-Neu5Ac, cytidine monophosphate sialic acid; UDP-Glc, UDP-glucose; UDP-Gal, UDP-galactose; UDP, uridine diphosphate.

Although the *lic2A* gene contributes positively to serum resistance under some conditions, deletion of *lic2A* restores serum resistance and improves lung colonization of *galE* mutants. Conditions in which *galE* activity is absent or low in wild-type NTHi have not been studied extensively, however the NTHi *galE* mutant exhibits enhanced production of biofilm suggesting that biofilm formation may involve decreased GalE activity (Greiner et al., 2004). We noticed in our HITS data that several genes required in the lung model in the *lic2A* mutant but not in the parent have been implicated in promoting biofilm formation, including genes of phosphate homeostasis (*phoB, pstA*) (Monds et al., 2001; Hsieh and Wanner, 2010; Xu et al., 2012); stress adaptation (*lon, sspA, deaD, aspA*) (Iost and Dreyfus, 2006; Marr et al., 2007; Van Melderen and Aertsen, 2009; Nijland and Burgess, 2010; Bernier et al., 2011; Giaouris et al., 2013; Karatan and Michael, 2013; Vakulskas et al., 2014; Rogers et al., 2016; Xie et al., 2016; Ching et al., 2018), and DNA replication, recombination, and repair (*ihfA, xerC, fis*) (Barre et al., 2001; Sheikh et al., 2001; Goodman et al., 2011; Moor et al., 2014; Atwood et al., 2016; Leite et al., 2017; Devaraj et al., 2018; Lv et al., 2018; Supplementary Text S1). Together, these observations suggest that during NTHi pathogenesis, biofilm formation may involve a switch to decreased reliance on LOS production. Such a switch could be induced by metabolites that NTHi scavenge from the host. Sialic acid has been shown to enhance NTHi biofilm production and both sialic acid and possibly *N*-acetylglucosamine (GlcNAc) are present in biofilm matrix (Greiner et al., 2004; Swords et al., 2004). Both GlcNAc and sialic acid are precursors of UDP-GlcNAc (Figure 13A), substrate donor for synthesis of poly-*N*-acetyl-glucosamine, a potential component of biofilm matrix that has been detected on the NTHi cell surface (Cywes-Bentley et al., 2013). Because likely orthologs of GalE of NTHi in other species can utilize UDP-GlcNAc as an alternative substrate (see Discussion), we postulate that exogenous GlcNAc/sialic acid in the environment could shift NTHi from UDP-Gal production toward synthesis of UDP-GlcNAc, leading to increased biofilm production. To begin to evaluate this hypothesis we conducted biofilm assays with NTHi 375 and isogenic *galE* and *siaB* mutants in the presence of these metabolites. The results showed that addition of exogenous GlcNAc and sialic acid fueled biofilm production in the wild type and in the *galE* mutant (Figure 13B). As previously shown, the asialylated *siaB* mutant exhibited reduced biofilm formation (Greiner et al., 2004; Swords et al., 2004). We note that exogenous galactose bi-passes the role of GalE, as enhanced biofilm production induced by sialic acid or GlcNAc is abrogated in the *galE* mutant by exogenous galactose. However, exogenous galactose did not influence biofilm production in the presence of both GlcNAc and sialic acid in the wild type, which synthesizes galactose endogenously.

## Discussion

We conducted a genome-wide genetic interaction screen to compare fitness of transposon mutants in a *lic2A* deletion background to that of the same mutants in the parental strain background, and thereby identify mutants with virulence phenotypes that are exacerbated by deletion of *lic2A*. This approach can identify genes that become required in natural phase variants in the *lic2A* off-phase, and also address the previously observed discrepancy between the virulence phenotypes of *galE* and *lic2A* mutants. A large number of mutations conferring virulence defects in the *lic2A* deletion background were identified in the screen, including mutations in known or putative LOS glycosyltransferase genes, a primary class we had anticipated finding. Surprisingly, the *galE* mutant exhibited a prominent phenotype in that its survival defect in the lung model was suppressed by deletion of *lic2A*. A major defect in serum resistance in the *galE* mutant was shown to result from an alternative epitope dependent on the presence of a functional *lic2A* gene but produced only when *galE* is inactivated. We found that serum IgM targets this epitope, leading to killing of NTHi by the classical pathway of complement. Therefore, a major virulence function of *galE* is to prevent alternative substrate utilization by the glycosyltransferase encoded by *lic2A*, which leads to generation of a novel IgM target and complement sensitivity. Nevertheless, the *lic2A* gene can also contribute positively to pathogenesis, as highlighted by survival defects in the lung model detected with transposon insertion mutations in a diversity of other genes in the *lic2A* deletion background. These genes are likely to be important in *lic2A* phase variants. We also cannot rule out a potential virulence role for this alternative Lic2A activity in some aspect of infection not reflected in the lung model. Finally, a question remains as to when UDP-Gal production by GalE might be decreased under natural conditions, a situation that could account for the evolution of phase-variation by *lic2A* as a means of preventing inappropriate epitope production. In this report, we suggest that the latter may occur in biofilms, as *galE* mutants exhibit increased biofilm formation, and host metabolites that increase biofilm production are also likely modulators of substrate utilization by GalE.

Deletion of *lic2A* generated or increased the requirement for numerous genes involved in host-pathogen interactions. Almost all of the *vacJ*/*mla* (*yrb)* homologs that function in phospholipid trafficking to maintain lipid asymmetry in the outer membrane were implicated (Malinverni and Silhavy, 2009). In NTHi, *vacJ, yrbE, yrbB*, and *yrbD* mutants are very sensitive to NHS (Nakamura et al., 2011), which may magnify sensitivity in the *lic2A* deletion background to killing by complement. We also observed the requirement for *dacA* (*pbp5*), which is involved in peptidoglycan biosynthesis and *HI1658* (*yraP*), a predicted outer membrane lipoprotein that is a component of the divisome (Tsang et al., 2017) and contributes to maintaining the outer membrane barrier (Morris et al., 2018). The requirement for *lic1*, responsible for LOS modification with phosphorylcholine, is likely due to increased IgM binding of the *lpsA* dependent LOS structure exposed in a *lic2A lic1* double mutant that was correlated with increased neutrophil-mediated killing in a previous study (Langereis and Weiser, 2014). We anticipated identifying LOS glycosyltransferase candidates such as the genes (*hmg*) required for the cryptic LOS glycoforms or genes (*lsg*) that associate with this structure (Hood et al., 2004b). Although, we did not find a requirement for *hm*g in the *lic2A* mutant background for *in vivo* survival, the *lsg* glycosyltransferase genes did appear to be functionally redundant with *lic2A in vivo*. But since the *lsg* mutations in the *lic2A* deletion background did not lead to increased killing compared to the *lic2A* mutation alone in the serum bactericidal assays, functional involvement of *lsg* genes with *lic2A* in pathogenesis is currently unclear, and may involve phenotypes other than complement resistance.

The most striking finding from our genetic screen is that *galE*, which supplies the precursor sugar for LOS incorporation by Lic2A, was not required for survival in the lung in the *lic2A* deletion strain. We identified a *lic2A* dependent immune target leading to serum sensitivity of the *galE* mutant and its attenuation *in vivo*. We hypothesize that the epitope added by *lic2A* in the absence of *galE* likely results from addition of a second glucose onto that added by *lpsA* on HepIII of the LOS. Structural analyses of LOS in *H. influenzae* type b strain Eagan indicate a galactose residue is normally attached by *lic2A* in a β1,4 linkage to a diglucoside on the middle HepII residue to form a Gal-Glc-Glc trisaccharide (Masoud et al., 1997; Hood et al., 2001, 2004a). However, both Eagan *galE* and double *galE galK* mutants display a novel triglucoside chain extension off HepII not normally found in *H. influenzae* LOS (Masoud et al., 2008), which is consistent with addition of Glc by Lic2A in the absence of UDP-Gal. In fact, it has been shown that there is some flexibility in the donor specificity of galactosyltransferases. For example, bovine β1,4-galactosyltransferase does not have an absolute requirement for UDP-Gal as donor, but at a lower efficiency can use other donors, such as UDP-Glc (Palcic and Hindsgaul, 1991). The amino acid sequence of the Rd Lic2A has 96 and 98% identities to Hib and NTHi 375 Lic2A, respectively, and they likely function similarly amongst diverse strains. Interestingly, reports in *Neisseria meningitides* indicated that a *galE* mutant has the expected major LOS species lacking galactose, but also displays a second LOS species (5–10% of the total) containing an extra glucose linked to the proximal glucose of the outer core (Lee et al., 1995; Wakarchuk et al., 1996). This extra glucose is dependent on the *lgtE* gene, whose product normally functions as a galactosyltransferase.

We speculate that a critical role of GalE in *H. influenzae* is to prevent Lic2A from adding an immune target, presumably a glucose extension, in the absence of a galactose source. Because the *lic2A* gene is subject to phase variation and can randomly and reversibly switch to the phase-off state, GalE activity in this scenario could decrease without triggering an adverse immune response, and adapting to niches that cause low GalE activity may be one of the roles of phase variation of *lic2A*. While such conditions have not been identified for NTHi *in vivo*, several of our results point to the biofilm mode of growth as a potential modulator of GalE, as discussed below. Biofilm formation by NTHi is known to occur in persistent infections in the airways (Swords, 2012) and has been found in the middle ear mucosa in a chinchilla experimental model of otitis media (OM) (Ehrlich et al., 2002) and in middle ear effusions in children with OM (Idicula et al., 2016).

We note that several genes required in the *lic2A* deletion background (Table 2) have potential orthologs in other bacterial species that participate in biofilm formation, such as *phoB* and *pstA* of the phosphate regulon; stress response/adaptation genes, *lon, sspA, deaD*, and *aspA*, and DNA metabolism genes, *ihfA, xerC*, and *fis*. In NTHi, the DNA binding protein IHF was found to be important in maintaining structural integrity of the extracellular DNA component of biofilm and treatment with anti-IHF could destabilize NTHi biofilms *in vitro* and *in vivo* in chinchilla middle ears (Goodman et al., 2011; Devaraj et al., 2018). Additional description of these genes is provided in Supplementary Text S1.

It is intriguing to speculate that a consequence of *lic2A* phasing off is an alleviated requirement for *galE* expression as part of adaptation within a biofilm. Because mutants lacking *galE* produce enhanced biofilm, it is possible that reduced UDP-Gal production by GalE may occur in biofilms. While sialic acid and at least one glycosyltransferase have been implicated in biofilm production (Pang et al., 2018), the exact composition of the carbohydrates in the matrix is not known. Nevertheless, cell surface poly-*N*-acetylglucosamine (PNAG), is a major polysaccharide component of biofilm matrix in other bacteria (Roux et al., 2015), and the precursor for PNAG, UDP-GlcNAc, has been proposed as a precursor of biofilm matrix material in NTHi (Greiner et al., 2004). Moreover, PNAG was recently detected on the NTHi cell surface (Cywes-Bentley et al., 2013), although the genes responsible for PNAG biosynthesis in NTHi have not been identified.

To begin to evaluate the hypothesis that biofilm formation may involve metabolic flux away from UDP-Gal production and toward higher UDP-GlcNAc levels, we conducted biofilm assays with precursors of this sugar nucleotide. Our biofilm assay results showed a statistically significant ~2-fold increase in biofilm formation in the NTHi 375 wild-type and *galE* mutant when grown in the presence of both GlcNAc and sialic acid supplied together (dark green bars) vs. sBHI alone (white bars) (Figure 13B), consistent with a potential role in biofilms for UDP-GlcNAc, which is generated from both of these substrates (Mengin-Lecreulx and Van Heijenoort, 1993, 1996; Figure 13A). The ~2-fold increase in biofilm formation in the *galE* mutant vs. wild type in sBHI alone is likely due to traces of sialic acid in sBHI as sialic acid is known to induce biofilm formation (Greiner et al., 2004; Swords et al., 2004).

In the NTHi 375 wild-type, GlcNAc or sialic acid alone did not enhance biofilm formation as much as it did in the *galE* mutant, and biofilm formation overall was higher in the *galE* mutant. These results could be accounted for if GalE in the wild-type strain has the capacity to interconvert UDP-GlcNAc to UDP-GalNAc (Figure 13A), providing GalNAc for LOS biosynthesis and thereby reducing levels UDP-GlcNAc available for biofilm formation, potentially involving PNAG production. GalE is a candidate for mediating this conversion as *H. influenzae* LOS contains GalNAc residues (Risberg et al., 1999; Hood et al., 2004b), yet there is no annotated UDP-GlcNAc 4-epimerase gene (*gne*) in its genome. In bacteria that do not possess *gne* homologs, such as *Campylobacter jejuni* and *Neisseria* spp, UDP-GlcNAc is synthesized by a bi-functional GalE epimerase (Bernatchez et al., 2005; Bartley et al., 2017). A single amino acid in GalE determines substrate specificity for UDP-Gal/Glc (mono-functional) vs. both UDP-Gal and UDP-GalNAc/GlcNAc (bi-functional) (Thoden et al., 2002; Bartley et al., 2017). Interestingly, a BLAST database search of GalE of 62 complete genome sequences of *H. influenzae* strains examined indicates that all possess a cysteine, C299, corresponding to the critical substrate specificity site, Y299, of *E. coli* GalE, in which a Y299C mutation converts it from a mono-functional to a bi-functional enzyme (Thoden et al., 2002). It is intriguing to speculate that GalE in *H. influenzae* may possess bi-functional activity and that this activity could potentially be modulated by UDP-GlcNAc precursors affecting biofilm production.

Aside from *galE*, the genetic screen also identified *xthA* and *HI1146* as dispensable in the *lic2A* mutant but required in the parent. XthA (exodeoxyribonuclease III) participates in repair of oxidative damage to DNA (Lovett, 2011), resistance to oxidative stress (Demple et al., 1983) and resistance to hydrostatic pressure during stationary phase (Charoenwong et al., 2011). It is not clear why deletion of *lic2A* would alleviate these mutant phenotypes in *H. influenzae*. *HI1146* encodes a putative RapZ (or YhbJ) homolog (RNase adaptor protein) that functions in a small RNA mediated regulatory feedback pathway, inhibiting expression of glucosamine-6-phosphate synthase (GlmS) in response to its product, glucosamine 6-phosphate (GlcN-6P), a key intermediate for the biosynthesis of UDP-GlcNAc and UDP-GalNAc (Figure 13A; Kalamorz et al., 2007; Göpel et al., 2013). Therefore, deletion of the putative *rapZ* homolog *HI1146*, is predicted to increase intracellular GlcN-6P, which may shift cellular production away from UDP-Gal and toward UDP-GlcNAc, leading to a Lic2A dependent virulence defect similar to that seen in a *galE* deletion mutant.

Our findings indicate that when deprived of its usual galactose donor substrate provided by GalE or the exogenous pathway, the *lic2A* encoded galactosyltransferase generates an alternative epitope that is an IgM antibody immune target leading to increased serum sensitivity and attenuation in the lung. Because virulence in a wide range of bacterial pathogens depends upon GalE, its role for NTHi in controlling alternative glycosyltransferase phenotypes identified herein has broad implications toward understanding its role in other bacterial pathogens of high clinical significance.

## Data Availability

Transposon insertion site sequencing (HITS) read data have been deposited in the NCBI short read archive (SRA) with the Bioproject ID number: PRJNA516677.

## Ethics Statement

Experiments were conducted with approval and in accordance with guidelines of the Institutional Animal Care and Use Committee at the University of Mississippi Medical Center (Jackson, MS).

## Author Contributions

SW and BA designed experiments, analyzed data and wrote the manuscript. SW performed all experiments with MJ contributing to studies of IgM binding, immunoblotting, serum bactericidal assays, and serum absorption studies. All authors read and approved the manuscript.

### Conflict of Interest Statement

The authors declare that the research was conducted in the absence of any commercial or financial relationships that could be construed as a potential conflict of interest.
