# Supplementary material for: Suppression of Alternative Lipooligosaccharide Glycosyltransferase Activity by UDP-Galactose Epimerase Enhances Murine Lung Infection and Evasion of Serum IgM
Source: Front Cell Infect Microbiol. 2019 May 15;9:160. doi: 10.3389/fcimb.2019.00160 (PMC6530457; doi:10.3389/fcimb.2019.00160)
Supplement: Supplementary file 5 [file Data_Sheet_1.PDF]

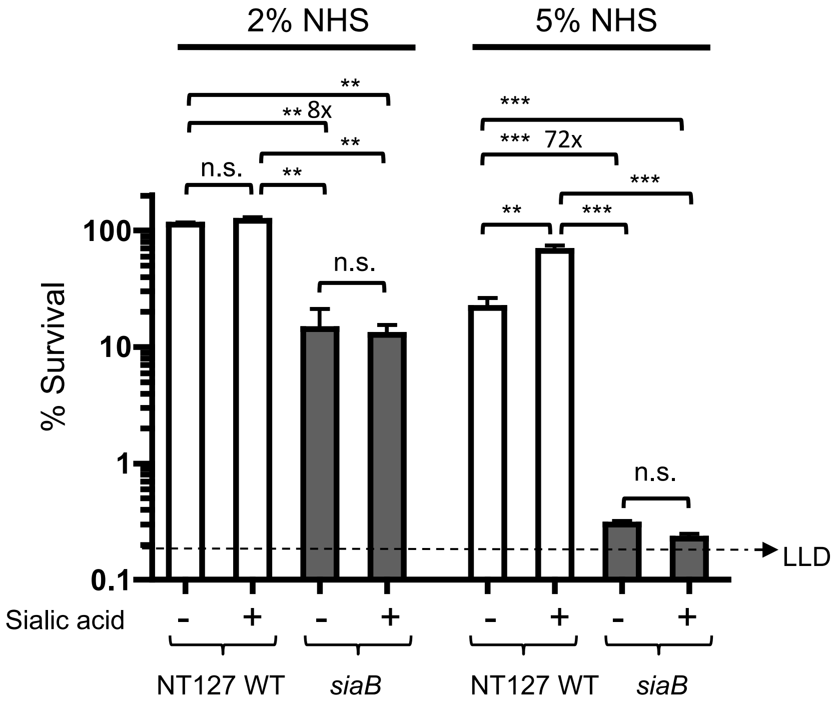

Figure S1. Serum resistance profile of the wild-type NTHi strain NT127 versus the *siaB* mutant confirms the presence of sialic acid in sBHI. Viability of NT127 WT and isogenic deletion mutant *siaB* grown in sBHI following incubation at 37ºC for 30 min with 2% and 5% NHS in the presence and absence of supplemental 50 µg/ml sialic acid. Percent survival is the ratio of CFU recovered at 30 min from samples treated with NHS to CFU recovered from untreated samples. Survival ratios were evaluated by one-way ANOVA with Bonferroni's multiple comparison test (**, $p < 0.01$; ***, $p < 0.001$; n.s., not statistically significant). The mean of triplicate samples is shown. LLD, lower limit of detection.
