# Supplementary material for: Suppression of Alternative Lipooligosaccharide Glycosyltransferase Activity by UDP-Galactose Epimerase Enhances Murine Lung Infection and Evasion of Serum IgM
Source: Front Cell Infect Microbiol. 2019 May 15;9:160. doi: 10.3389/fcimb.2019.00160 (PMC6530457; doi:10.3389/fcimb.2019.00160)
Supplement: Supplementary file 6 [file Data_Sheet_2.PDF]

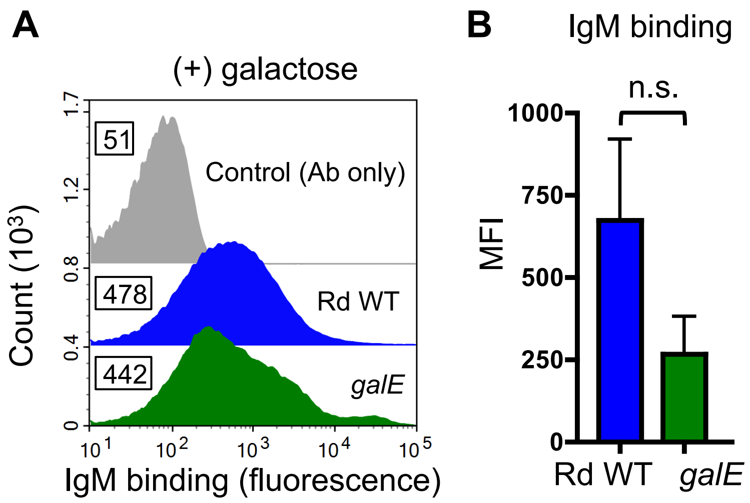

Figure S2. Increased IgM binding to the Rd *galE* mutant compared to wild-type does not occur in the presence of galactose. **(A)** Rd wild-type (WT) and *galE* mutant grown in MIc^{SA} in the presence of 0.5% galactose incubated with $NHS^{\Delta i}$ at 20% final concentration at 37°C for 30 min followed by detection via flow cytometry with anti-human IgM conjugated to FITC. X-axis; fluorescence; Y-axis, counts. Numbers alongside the histograms indicate the MFI of the bacterial population. Control antibody (Ab) only fluorescence values are similar for both strains and only one representative is shown (Rd WT). One representative experiment of two reproducible repeats is shown. **(B)** IgM binding for the mean of replicate samples of which one is shown in panel A. Difference between WT and the *galE* mutant was not statistically significant (n.s.) (unpaired *t*-test with Welch's correction).
