# Supplementary material for: Suppression of Alternative Lipooligosaccharide Glycosyltransferase Activity by UDP-Galactose Epimerase Enhances Murine Lung Infection and Evasion of Serum IgM
Source: Front Cell Infect Microbiol. 2019 May 15;9:160. doi: 10.3389/fcimb.2019.00160 (PMC6530457; doi:10.3389/fcimb.2019.00160)
Supplement: Supplementary file 7 [file Data_Sheet_3.PDF]

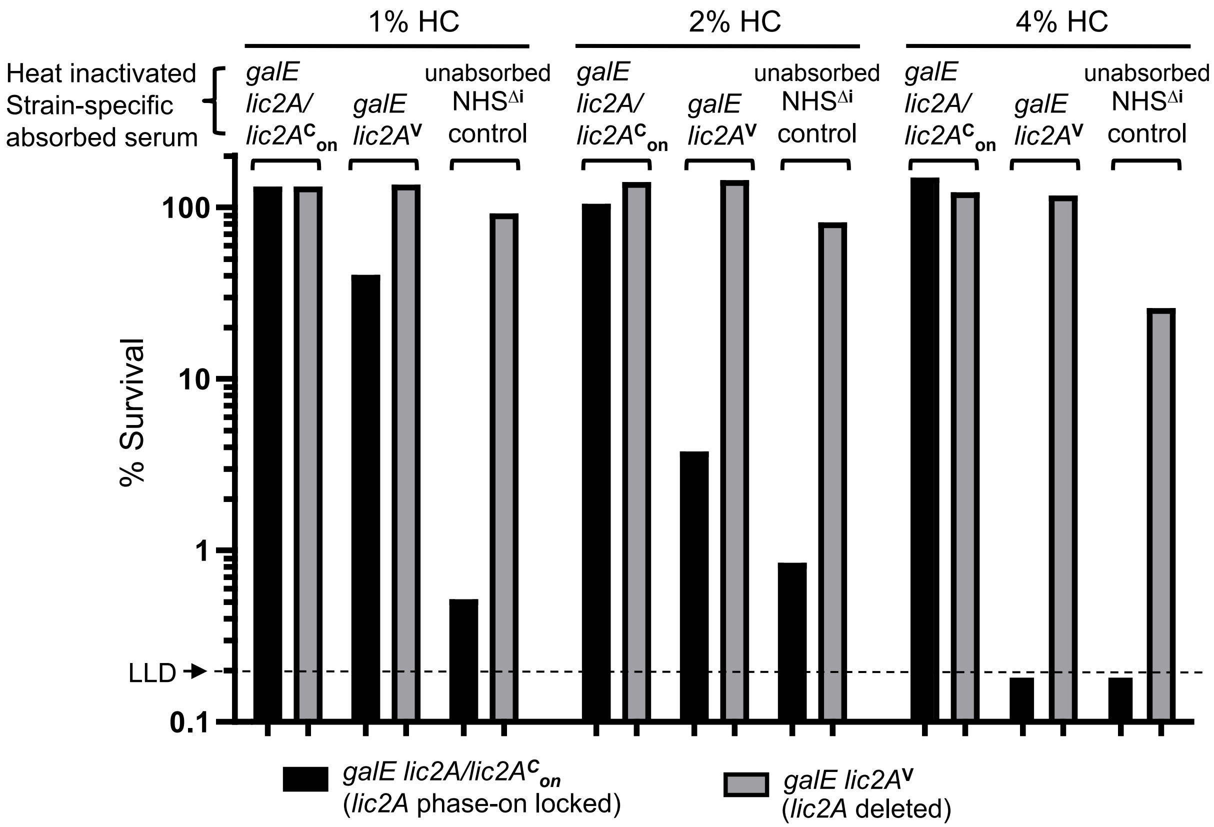

Figure S3. Absorption of serum with an Rd *galE* deficient strain expressing *lic2A* decreases antibody-dependent killing of this mutant by complement. Viability of Rd mutants *galE lic2A* containing a complementing copy of phase-on locked *lic2A in trans*, (*galE lic2A*/*lic2A*$^{C}_{on}$) and *galE lic2A* containing empty vector pXT10 in *xyl* (*galE lic2A*^{V}) grown on MIc^{SA} medium was assayed following incubation at 37°C for 5 min with 10% NHS$^{\Delta i}$ pre-absorbed with *galE lic2A*/*lic2A*$^{C}_{on}$ *or galE lic2A*^{V} or control unabsorbed NHS$^{\Delta i}$ prior to further incubation with 1%, 2% or 4% IgG/IgM antibody depleted human complement active serum (HC) at 37°C for 30 min. Percent survival is the ratio of CFU recovered from absorbed serum treated samples plus HC at 30 min to CFU recovered from untreated samples plus HC. Lower limit of detection is approximately 0.2% survival.
