# Supplementary material for: Suppression of Alternative Lipooligosaccharide Glycosyltransferase Activity by UDP-Galactose Epimerase Enhances Murine Lung Infection and Evasion of Serum IgM
Source: Front Cell Infect Microbiol. 2019 May 15;9:160. doi: 10.3389/fcimb.2019.00160 (PMC6530457; doi:10.3389/fcimb.2019.00160)
Supplement: Supplementary file 8 [file Data_Sheet_4.PDF]

Text S1

Methods

Plasmid and strain construction.

Primer sequences used in this study are listed in Table S1. A nonpolar in-frame deletion mutant of *lic2A* was created by replacement of the protein coding sequences with the *aacC1* gentamicin resistance cassette by polymerase chain reaction (PCR) stitching as follows: A 1045 bp PCR product containing the 5' flanking region of *lic2A* was amplified from Rd with primers 550-1kbup and 550-5out. A 1010 bp PCR product containing the 3' flanking region of *lic2A* was amplified from Rd with primers 550-3out and 550-1kbdown. A 536 bp fragment containing the *aacC1* gentamicin resistance gene was amplified with primers aacC15' and aacC13' from pBSL182 (Alexeyev and Shokolenko, 1995). The 1045 bp, 1010 bp, and 536 bp products were stitched in a PCR reaction with primers 550-1kbup and 550-1kbdown. The resultant ~2.5 kb product was introduced into Rd and Gm^R transformants were selected on sBHI agar containing Gm. A nonpolar, in-frame deletion mutant of *galE* in Rd is described previously (Wong et al., 2013). Similarly, *lic2A* and *galE* single deletion mutations in NTHi 375 were created by transforming this strain with the ~2.4 kb PCR product (above) and the ~2.8 kb *galE* knockout PCR product (Wong et al., 2013) and selecting for Gm^R or Km^R transformants on sBHI agar containing Gm or Km, respectively. The *galE* and *lic2A* double deletion mutant in NTHi 375 was created by introducing the ~2.4 kb *lic2A* knockout PCR product into the NTHi 375 *galE* mutant; the *galE* and *lic2A* double deletion mutant in Rd was created by introducing the ~2.8 kb *galE* knockout PCR product into the Rd *lic2A* deletion mutant. All strains used in this study, except for those with deletions in *lic2A,* were analyzed by PCR with primers 50550 and 30550 to amplify *lic2A*, and primers 550-5'caat and 550-3'caat to sequence the PCR products across the *lic2A* 5' regions containing the phase variable CAAT repeat tracts, verifying that the *lic2A* gene is phase-on, containing 22 repeats corresponding to the active open reading frame (Dixon et al., 2007).

Nonpolar in-frame deletion mutants of *lsgE*, *lsgD*, and *lsgC* in Rd were created by replacement of the protein coding sequences with the *ermC'* erythromycin resistance cassette under the synthetic *E. coli trc* promoter by PCR stitching with the initial step as

follows: A 735 bp fragment containing the *ermC'* gene was amplified with primers ermC5orf and ermC3orf from pFLOB4300 (Johnston and Cannon, 1999) (a kind gift of Drs. Lisa Lewis and Sanjay Ram) and stitched to a 264 bp PCR product containing the *trc* promoter from pTrcHIs2B (ThermoFisher Scientific) amplified with primers PTrc5CGA and PTrcstitchErm to yield a 984 bp product, TrcP-*ermC'*. To generate an *lsgC* mutant, a 931 bp PCR product containing the 5' flanking region of *lsgC* was amplified from Rd with primers 5-1699 and 1698-5out. A 1059 bp PCR product containing the 3' flanking region of *lsgC* was amplified from Rd with primers 1698-3out2 and 1698-1kb3. The 931 bp, 1059 bp, and the 984 bp (TrcP-*ermC'*) products were stitched in a PCR reaction with primers 5-1699 and 1698-1kb3 to yield the resultant ~3 kb *lsgC* knockout PCR product. To generate an *lsgD* mutant, an 1109 bp PCR product containing the 5' flanking region of *lsgD* was amplified from Rd with primers 5-1698-2 and 1697-5out. A 917 bp PCR product containing the 3' flanking region of *lsgD* was amplified from Rd with primers 1697-3out2 and 3-1696orf2. The 1109 bp, 917 bp, and 984 bp (TrcP-*ermC'*) products were stitched in a PCR reaction with primers 5-1698-2 and 3-1696orf2 to yield the resultant ~3 kb *lsgD* knockout PCR product. To generate an *lsgE* mutant, an 800 bp PCR product containing the 5' flanking region of *lsgE* was amplified from Rd with primers 1697-5'+1696-5out. A 1094 bp PCR product containing the 3' flanking region of *lsgE* was amplified from Rd with primers 1696-3out2+1696-1kb3. The 800 bp, 1094 bp, and 984 bp (TrcP-*ermC'*) products were stitched in a PCR reaction with primers 1697-5'and 1696-1kb3 to yield the resultant ~2.8 kb *lsgE* knockout PCR product. The resultant *lsg* knockout PCR products were introduced into Rd and Erm^R transformants were selected on sBHI agar containing Erm. The *lic2A lsg* double deletion mutants in Rd were created by introducing each *lsgE*, *lsgD*, and *lsgC* knockout PCR products (above) into the Rd *lic2A* deletion mutant and selecting for Erm^R transformants on sBHI agar containing Erm.

A nonpolar, in-frame deletion mutant of *galK* in Rd was created by replacement of the protein coding sequences with the *aacC1* gentamicin resistance cassette by PCR stitching as follows: A 997 bp PCR product containing the 5' flanking region of *galK* was amplified from Rd with primers 1kb5-HI0819 and HI0819-5outgent. A 982 bp PCR product containing the 3' flanking region of *galK* was amplified from Rd with primers HI0819-3outgent and 1kb3-HI0819. These two products and the 536 bp *aacC1* gene were

stitched in a PCR reaction with primers 1kb5-HI0819 and HI0819-5outgent. The resultant ~2.5 kb product was introduced into Rd and Gm^R transformants were selected on sBHI agar containing Gm. The *galE* and *galK* double deletion mutation in Rd was created by introducing the ~2.8 kb *galE* replacement PCR product into the *galK* deletion mutant.

Wild-type Rd strain containing the empty allelic exchange vector pXT10 (Wong and Akerley, 2003) (RdV, called here as Rd^V) was described previously (Wong et al., 2007). Rd strain, RdLacZ containing a xylose inducible *lacZ* gene integrated at the *xyl* locus was generated with plasmid pXELacZ2 as described (Rosadini et al., 2011). The single *lic2A* and *galE* mutants and the double *lic2A* and *galE* deletion mutants containing the empty cloning vector were created by transformation with pXT10 and selecting for transformants on sBHI agar containing Tet. The *galE* complementing strain in Rd was created as follows: A 1269 bp product containing the *galE* protein coding sequence and adjacent upstream promoter region were amplified from Rd with primers 5RdgalESap and 3RdgalESap. The PCR product was digested with *Sap*I and cloned into the *Sap*I sites of pXT10. This plasmid was introduced into the *xyl* locus of the *galE* deletion mutant and Tet^R transformants were selected on sBHI agar containing Tet. To create a Rd strain which contains a complementing copy of *lic2A* phase-on locked *in trans*, a construct containing a deletion of all of the CAAT tandem repeats except for one in the *lic2A* open reading frame was created as follows: A 264 bp PCR product containing the 5'region of *lic2A* was amplified from Rd with primers 375lic2A-5sap and Rd lic2A-5out. A 739 bp PCR product containing the 3'region of *lic2A* was amplified from Rd with primers 375 lic2A-3out and 375lic2A-3sap. The two products were stitched in PCR with primers 375lic2A-5sap and 375lic2A-3sap. The resultant ~990 bp product was digested with *Sap*I and cloned into the *Sap*I sites of pXT10. This construct was introduced into the *xyl* locus of the *galE* and *lic2A* double deletion mutant to create strain RdgalElic2A/$lic2A^C_{on}$ containing *lic2A* phase on locked. Δrep strain containing *licA* phase-on locked in Rd was described previously (Wong and Akerley, 2005). The *lic2A* deletion mutant in the Δrep background was created by transforming the ~2.5 kb *lic2A* knockout PCR product (above) into Δrep and selecting for Gm^R transformants on sBHI agar containing Gm to create Δreplic2A.

A nonpolar in-frame deletion mutant of *siaB* in clinical isolate NT127 (Wong et al., 2013) was created by replacement of the protein coding sequence with a kanamycin

resistance gene by PCR stitching as follows: A 1019 bp PCR product containing the 5' flanking region of *siaB* was amplified from NT127 with primers 1kb5'JBsiaB and JBsiaB-5'out. A 1023 bp PCR product containing the 3' flanking region of *siaB* was amplified from NT127 with primers JBsiaB-3'out and 1kb3'JBsiaB. An 818 bp fragment containing the kanamycin resistance gene, *aphI* from Tn*903* was amplified with primers kan5+ATG and kan3'+TAA (Wong et al., 2013). The 1019 bp, 1023 bp, and 818 bp products were stitched in a PCR reaction with primers 1kb5'JBsiaB and 1kb3'JBsiaB. The resultant ~2.8 kb product was introduced into NT127 and Km^R transformants were selected on sBHI agar containing Km. For NTHi 375 carrying the *siaB* mutation, we thank Dr. Derek Hood for the 375*siaB* strain (Bouchet et al., 2003).

Absorption of sera.

For absorption, 4 x 10^{10} each of strains RdgalElic2A/lic2A$^{C}_{on}$ and RdgalElic2A grown on MIc agar containing sialic acid (25 μg/ml) were harvested, washed in Hank's Balanced Salt Solution with calcium and magnesium chloride (HBSS++), pelleted and incubated with 1ml of NHS$^{\Delta i}$ was added to each pellet containing 10^{10} bacteria and incubated for 30 min at 37°C followed by centrifugation for 5 min at room temperature. Serum supernatant was collected and added to a fresh aliquot of 10^{10} bacteria with the absorption procedure repeated for a total of four rounds. At the final round, the bacterial suspension was incubated at 4°C overnight with gentle agitation prior to centrifugation to collect absorbed sera.

Serum bactericidal assays.

Serum bactericidal assays were performed as previously described (Wong et al., 2011) with ~3,000 CFU of bacteria incubated with or without NHS (Innovative Research) and NHS$^{\Delta i}$ at a concentrations specified for each experiment in a final reaction mixture volume of 150 μl in HBSS++/0.1%BSA for 30 min at 37°C. Dilutions of untreated samples were plated on sBHI agar plates at 0 min to verify similar numbers of input bacteria for each sample. Dilutions of NHS treated versus NHS$^{\Delta I}$ treated samples were plated on sBHI agar plates at 30 min. Survival was calculated as the ratio of CFU recovered from samples treated with either NHS or NHS$^{\Delta I}$ at 30 min to those from untreated samples (Figures 4 and S1), or in

other experiments as the ratio of CFU recovered at 30 min from samples treated with NHS to those with $NHS^{\Delta i}$. All experiments were conducted on duplicate or triplicate independent samples as indicated in figure legends. For bactericidal assays using strain specific absorbed sera, percent survival was calculated as the ratio of CFU recovered from $NHS^{\Delta i}$ treated vs. untreated (buffer only) samples after HC incubation.

***H. influenzae* colonization in the murine lung model.**

For single strain colonization assays and *in vivo* competition assays, *H. influenzae* strains were inoculated intranasally into mice and recovered from lungs at ~24 hr. post-bacterial inoculation. For *in vivo* competition assays, 10^7 CFU of Rd^V, $Rdlic2A^V$, $RdgalE^V$, RdgalE/$galE^C$, $RdgalElic2A^V$ and RdgalElic2A/$lic2A^C_{on}$ were co-inoculated with an equal number of competitor RdLacZ (expresses *lacZ*) into the nares of mice (n=5), and recovered CFU enumerated on sBHI with S-Gal. Ratios of CFU of the experimental strains (white colonies, LacZ-) to competitor strain (black colonies, LacZ+) were reported as the competitive index. For the single strain colonization assay, 10^7 CFU of NTHi 375 wild-type and isogenic mutants were inoculated into the nares of mice (n=10-12). Bacterial CFU were recovered and enumerated from lungs at ~ 24 hr. post-infection.

Sequencing of transposon junctions and analysis of sequence data.

After aligning the sequence reads to the *H. influenzae* Rd KW20 genome, GenBank: L42023.1 (National Center for Biotechnology Information, NCBI), assigning reads to genes and filtering for unique reads, read counts obtained for in *vitro* libraries and output libraries recovered from mice were 1,733,828 and 1,790,931 (parent library); 652,601 and 2,399,822 (*lic2A* library), respectively. 1,416,631 and 538,141 sequencing reads were in protein coding genes with 317,197 and 114,460 reads in RNA structural genes or intergenic regions for parent and *lic2A* libraries, respectively. The average ratios of the number of output reads divided by the input reads in each gene were determined. After trimming the 15% highest and lowest ratios the median ratios were used to obtain normalization factors of 0.925 and 3.05, which were used to adjust the number of input reads from parent library and *lic2A* library, respectively. Student *t*-test *p* values were calculated from normalized unique input reads vs. unique output reads at each TA position in each gene. A fitness

survival index (s.i.) for a mutant after selection was calculated by dividing the total reads within the first 95% of the coding region of each gene from the output library by the total reads (normalized) from the same region in the input library.

Of the 1,724 predicted or annotated genes in the genome, we excluded loci required for growth *in vitro* on rich medium (sBHI) from our analysis. These genes were identified with the Essential Loci analysis (EL-ARTIST) component of the Analysis of high-Resolution Transposon-Insertion Sequences Technique (ARTIST) pipeline (Pritchard et al., 2014) using MATLAB computing software (MathWorks, Inc., Natick, MA). EL-ARTIST analysis was performed on *in vitro* grown transposon library created in Δrep and Δreplic2A strains using a sliding window size of 7 (7 consecutive TA sites) and *p* value thresholds of 0.045 (for the parent Δrep library) and 0.04 (for the Δreplic2A library) as parameters for a region to be considered significantly underrepresented in reads in order to generate a list of sliding window essential regions for hidden Markov modeling (HMM) to refine assignment of essentiality. The *p* value parameters were determined based on the threshold yielding the highest stringency required for the sliding window analysis to identify non-essential and essential regions using this recommended sliding window size with the level of saturation of our transposon insertion data in each library. Final HMM predictions for functional categorization of each locus were: non-essential for growth; essential for optimal growth; domain essential (contains both regions that are required and dispensable for growth).

EL-ARTIST assigned 443 and 481 essential genes from the parent and *lic2A* input libraries, respectively that were excluded from our analysis. These two sets of essential genes had concordances of 96% and 90%, respectively compared to our previous HITS data in which we excluded genes based on essentiality (no insertions detected), near essentiality (insertions in <5% of their possible TA sites), inferred growth defects *in vitro* (insertions in >5% but less than <35% of their possible TA sites) and small gene size (<8 possible TA insertion sites) combined (Wong et al., 2013). From the parent library and *lic2A* library datasets, we further excluded 40 genes that contained sequences duplicated in the genome, genes with ≤ 5 read counts and genes with insertions in only one TA site within the first 95% of their coding regions (5 genes for parent library; 6 genes for *lic2A* library) leaving the remaining 1236 and 1197 genes, respectively to be further analyzed for their roles in fitness. A survival index < 0.2 and $p < 0.001$, corresponding to false discovery

rates (FDR) (Noble, 2009) of 1.2% and 1.3% for parent library and *lic2A* library, respectively, were set as requirements for genes to be considered to contribute to fitness during infection in the lung.

Discussion (additional): **Genes required in the *lic2A* deletion background.**

Our genetic interaction screen using HITS showed that several of the genes required in the *lic2A* deletion background (Table 1) have potential orthologs in other species that participate in biofilm formation. For example, the response regulator, *phoB* and ABC transporter permease, *pstA* of the phosphate regulon that function to maintain phosphate homeostasis (Hsieh and Wanner, 2010) have been reported to stimulate or enhance biofilm formation in the plant pathogen *Agrobacterium tumefaciens* (Xu et al., 2012) and *Pseudomonas aureofaciens* (Monds et al., 2001), respectively. Several genes had roles in stress response or adaptation (e.g. *lon*, *sspA*, *deaD*, *aspA*). The Lon protease, responsible for damaged protein turnover was also associated with responses to stress (DNA damage, acid tolerance, starvation), virulence phenotypes (Van Melderen and Aertsen, 2009) and was essential for biofilm development/formation in *Pseudomonas aeruginosa* (Marr et al., 2007), *Actinobacillus pleuropneumoniae* (Xie et al., 2016), *Vibrio cholerae* (Rogers et al., 2016) and *Acinetobacter baumannii* (Ching et al., 2018). The transcriptional activator SspA which is induced by starvation/stress was one of several proteins detected solely during biofilm growth vs. planktonic growth conditions in *Salmonella enterica* (Giaouris et al., 2013). *deaD* (or *csdA*) encodes a RNA helicase belonging to a family of helicases with conserved amino acids motifs including the DEAD-box involved in RNA metabolism (ribosome biogenesis, translation, mRNA decay) (Iost and Dreyfus, 2006). Usually *deaD*/*csdA* functions in adaptation to cold conditions, but was shown to operate at a higher temperature in regulating the carbon storage regulatory system in *E. coli* to promote biofilm formation (Vakulskas et al., 2014). *aspA* catalyzes the deamination of L-aspartate into fumarate and in the process produces ammonia as a by-product. Sensing of ammonia as a volatile compound produced from neighboring bacterial species cultured in adjacent microtiter wells induced biofilm formation in *Bacillus licheniformis* (Nijland and Burgess, 2010). Similarly, aerial exposure of *E. coli* by ammonia increased intracellular levels of polyamine enhancing resistance to oxidative stress (Bernier et al., 2011) and promoting

biofilm formation in *V. cholerae*, *Yersinia pestis*, *Bacillus subtilis*, *E. coli*, and *Neisseria gonorrhoeae* (Karatan and Michael, 2013).

Several genes with functions in DNA replication, recombination, and repair (*ihfA*, *xerC*, *fis*) that were also required in the *lic2A* mutant have been linked to biofilm formation. The XerC recombinase is required for chromosomal segregation at cell division (Barre et al., 2001) and a mutation in this gene reduced biofilm formation *in vitro* in a methicillin resistant strain of *Staphylococcus aureus* (Atwood et al., 2016). Fis and IhfA are DNA-binding proteins with global regulatory roles. Fis transcriptionally regulates diverse virulence traits and promotes biofilm formation in various gram negative bacteria including a pathogenic strain of *E. coli* (Sheikh et al., 2001), plant pathogen *Dickeya zeae* (Lv et al., 2018), and soil bacterium *Pseudomonas putida* (Moor et al., 2014). IhfA and IhfB mutants in *Salmonella enterica* were impaired in biofilm biomass and thickness (Leite et al., 2017).

References

Alexeyev, M.F., and Shokolenko, I.N. (1995). Mini-Tn*10* transposon derivatives for insertion mutagenesis and gene delivery into the chromosome of gram-negative bacteria. *Gene* 160, 59-62.

Atwood, D.N., Beenken, K.E., Loughran, A.J., Meeker, D.G., Lantz, T.L., Graham, J.W., Spencer, H.J., and Smeltzer, M.S. (2016). XerC Contributes to Diverse Forms of *Staphylococcus aureus* Infection via agr-Dependent and agr-Independent Pathways. *Infection and Immunity* 84, 1214-1225.

Barre, F.X., Soballe, B., Michel, B., Aroyo, M., Robertson, M., and Sherratt, D. (2001). Circles: the replication-recombination-chromosome segregation connection. *Proceedings of the National Academy of Sciences of the United States of America* 98, 8189-8195.

Bernier, S.P., Letoffe, S., Delepierre, M., and Ghigo, J.M. (2011). Biogenic ammonia modifies antibiotic resistance at a distance in physically separated bacteria. *Molecular Microbiology* 81, 705-716.

Bouchet, V., Hood, D.W., Li, J., Brisson, J.R., Randle, G.A., Martin, A., Li, Z., Goldstein, R., Schweda, E.K., Pelton, S.I., Richards, J.C., and Moxon, E.R. (2003). Host-derived sialic acid is incorporated into *Haemophilus influenzae* lipopolysaccharide and is a major virulence factor in experimental otitis media. *Proc Natl Acad Sci U S A* 100, 8898-8903.

Ching, C., Yang, B., Onwubueke, C., Lazinski, D., Camilli, A., and Godoy, V.G. (2018). Lon protease has multifaceted biological functions in *Acinetobacter baumannii*. *Journal of Bacteriology*.

Dixon, K., Bayliss, C.D., Makepeace, K., Moxon, E.R., and Hood, D.W. (2007). Identification of the functional initiation codons of a phase-variable gene of

Haemophilus influenzae, *lic2A*, with the potential for differential expression. *Journal of Bacteriology* 189, 511-521.

Giaouris, E., Samoilis, G., Chorianopoulos, N., Ercolini, D., and Nychas, G.J. (2013). Differential protein expression patterns between planktonic and biofilm cells of *Salmonella enterica* serovar Enteritidis PT4 on stainless steel surface. *International Journal of Food Microbiology* 162, 105-113.

Hsieh, Y.J., and Wanner, B.L. (2010). Global regulation by the seven-component Pi signaling system. *Current Opinion in Microbiology* 13, 198-203.

Iost, I., and Dreyfus, M. (2006). DEAD-box RNA helicases in *Escherichia coli*. *Nucleic Acids Res* 34, 4189-4197.

Johnston, D.M., and Cannon, J.G. (1999). Construction of mutant strains of *Neisseria gonorrhoeae* lacking new antibiotic resistance markers using a two gene cassette with positive and negative selection. *Gene* 236, 179-184.

Karatan, E., and Michael, A.J. (2013). A wider role for polyamines in biofilm formation. *Biotechnol Lett* 35, 1715-1717.

Leite, B., Werle, C.H., Carmo, C.P.D., Nobrega, D.B., Milanez, G.P., Culler, H.F., Sircili, M.P., Alvarez-Martinez, C.E., and Brocchi, M. (2017). Integration host factor is important for biofilm formation by *Salmonella enterica* Enteritidis. *Pathog Dis* 75.

Lv, M., Chen, Y., Liao, L., Liang, Z., Shi, Z., Tang, Y., Ye, S., Zhou, J., and Zhang, L. (2018). Fis is a global regulator critical for modulation of virulence factor production and pathogenicity of *Dickeya zeae*. *Sci Rep* 8, 341.

Marr, A.K., Overhage, J., Bains, M., and Hancock, R.E. (2007). The Lon protease of *Pseudomonas aeruginosa* is induced by aminoglycosides and is involved in biofilm formation and motility. *Microbiology* 153, 474-482.

Monds, R.D., Silby, M.W., and Mahanty, H.K. (2001). Expression of the Pho regulon negatively regulates biofilm formation by *Pseudomonas aureofaciens* PA147-2. *Molecular Microbiology* 42, 415-426.

Moor, H., Teppo, A., Lahesaare, A., Kivisaar, M., and Teras, R. (2014). Fis overexpression enhances *Pseudomonas putida* biofilm formation by regulating the ratio of LapA and LapF. *Microbiology* 160, 2681-2693.

Nijland, R., and Burgess, J.G. (2010). Bacterial olfaction. *Biotechnol J* 5, 974-977.

Noble, W.S. (2009). How does multiple testing correction work? *Nature Biotechnology* 27, 1135-1137.

Pritchard, J.R., Chao, M.C., Abel, S., Davis, B.M., Baranowski, C., Zhang, Y.J., Rubin, E.J., and Waldor, M.K. (2014). ARTIST: high-resolution genome-wide assessment of fitness using transposon-insertion sequencing. *PLoS Genet* 10, e1004782.

Rogers, A., Townsley, L., Gallego-Hernandez, A.L., Beyhan, S., Kwuan, L., and Yildiz, F.H. (2016). The LonA Protease Regulates Biofilm Formation, Motility, Virulence, and the Type VI Secretion System in *Vibrio cholerae*. *Journal of Bacteriology* 198, 973-985.

Rosadini, C.V., Gawronski, J.D., Raimunda, D., Arguello, J.M., and Akerley, B.J. (2011). A novel zinc binding system, ZevAB, is critical for survival of nontypeable *Haemophilus influenzae* in a murine lung infection model. *Infect Immun* 79, 3366-3376.

Sheikh, J., Hicks, S., Dall'agnol, M., Phillips, A.D., and Nataro, J.P. (2001). Roles for Fis and YafK in biofilm formation by enteroaggregative *Escherichia coli*. *Molecular Microbiology* 41, 983-997.

Vakulskas, C.A., Pannuri, A., Cortes-Selva, D., Zere, T.R., Ahmer, B.M., Babitzke, P., and Romeo, T. (2014). Global effects of the DEAD-box RNA helicase DeaD (CsdA) on gene expression over a broad range of temperatures. *Molecular Microbiology* 92, 945-958.

Van Melderen, L., and Aertsen, A. (2009). Regulation and quality control by Lon-dependent proteolysis. *Research in Microbiology* 160, 645-651.

Wong, S.M., and Akerley, B.J. (2003). Inducible expression system and marker-linked mutagenesis approach for functional genomics of *Haemophilus influenzae*. *Gene* 316, 177-186.

Wong, S.M., and Akerley, B.J. (2005). Environmental and genetic regulation of the phosphorylcholine epitope of *Haemophilus influenzae* lipooligosaccharide. *Mol Microbiol* 55, 724-738.

Wong, S.M., Alugupalli, K.R., Ram, S., and Akerley, B.J. (2007). The ArcA regulon and oxidative stress resistance in *Haemophilus influenzae*. *Mol Microbiol* 64, 1375-1390.

Wong, S.M., Bernui, M., Shen, H., and Akerley, B.J. (2013). Genome-wide fitness profiling reveals adaptations required by *Haemophilus* in coinfection with influenza A virus in the murine lung. *Proceedings of the National Academy of Sciences of the United States of America* 110, 15413-15418.

Wong, S.M., St Michael, F., Cox, A., Ram, S., and Akerley, B.J. (2011). ArcA-regulated glycosyltransferase *lic2B* promotes complement evasion and pathogenesis of nontypeable *Haemophilus influenzae*. *Infect Immun* 79, 1971-1983.

Xie, F., Li, G., Zhang, Y., Zhou, L., Liu, S., Liu, S., and Wang, C. (2016). The Lon protease homologue LonA, not LonC, contributes to the stress tolerance and biofilm formation of *Actinobacillus pleuropneumoniae*. *Microbial Pathogenesis* 93, 38-43.

Xu, J., Kim, J., Danhorn, T., Merritt, P.M., and Fuqua, C. (2012). Phosphorus limitation increases attachment in *Agrobacterium tumefaciens* and reveals a conditional functional redundancy in adhesin biosynthesis. *Research in Microbiology* 163, 674-684.
